# Toxic Trace Element Levels in Maternal and Cord Blood Worldwide and Their Impact on Pregnancy: A Narrative Review

**DOI:** 10.3390/jox16040132

**Published:** 2026-07-14

**Authors:** Radomir Aničić, Dejan Mihajlović, Jovana Kocić, Aleksandar Stojsavljević

**Affiliations:** 1 Faculty of Medicine, University of Belgrade, 11000 Belgrade, Serbia; radomir.anicic@gmail.com; 2 Department for Gynecology and Obstetrics, Clinical Health Center Kosovska Mitrovica, 38220 Kosovska Mitrovica, Serbia; 3 Clinic for Gynecology and Obstetrics “Narodni Front”, 11000 Belgrade, Serbia; gasicjovana@gmail.com; 4 Innovative Centre of the Faculty of Chemistry, University of Belgrade, 11000 Belgrade, Serbia; aleksandars@chem.bg.ac.rs

**Keywords:** toxic trace element, low exposure level, maternal and cord blood, demographics and lifestyles, normal and pathological pregnancy

## Abstract

Background: This up-to-date narrative review examines the associations of low environmental exposure to toxic trace elements—arsenic (As), cadmium (Cd), lead (Pb), and mercury (Hg)—on pregnancy. While high levels are known to be harmful, the impact of low levels has not been fully analyzed. The aim was to summarize global data on As, Cd, Pb, and Hg levels in maternal and cord blood of healthy pregnant women, compare their distribution, and assess associations with demographic factors, lifestyle, and pregnancy outcomes. Methods: A systematic literature search was conducted in PubMed, Cochrane Library, and Scopus for studies published between 1 January 1990 and 14 May 2025. The review was conducted in accordance with PRISMA 2020 guidelines. A total of 824 records were assessed for eligibility, and 656 were excluded based on predefined criteria. Exclusion criteria encompassed in vivo or in vitro studies, non-English written publications, treatment-based studies, and studies involving occupationally exposed pregnant women. Inclusion criteria included original full-length research papers with cross-sectional, prospective cohort, or case–control designs; studies measuring As, Cd, Pb, and/or Hg in maternal and/or cord blood; and studies examining associations with demographic, lifestyle, and pregnancy outcomes. The review was not registered, and no external funding was received. Given the narrative synthesis approach of the review, a formal risk-of-bias assessment was not undertaken. Results: A total of 168 studies were included: 32 reported findings on As, 55 on Cd, 78 on Pb, and 64 on Hg, with 26 addressing all four elements. No meta-analysis was performed; results were summarized narratively. Results show that As and Cd levels are higher in maternal blood, whereas Pb and Hg, including methylmercury, are higher in cord blood. Key factors include smoking, rural residence, and fish or seafood consumption, linked to higher Cd, Pb, and Hg levels, respectively. Interpretation: Low Pb levels show the strongest associations with adverse pregnancy outcomes, while As shows the weakest. Pb’s effects may relate to passive placental diffusion, unlike other elements. Further studies are needed.

## 1. Introduction

Among the vast number of pollutants in the environment, toxic trace elements pose one of the most significant health concerns, primarily due to their non-degradable nature and ability to remain in the human body for years. Arsenic (As), cadmium (Cd), lead (Pb), and mercury (Hg) are the [1] toxic trace elements that can harm human health [2]. None of these four elements is needed by the body, even in ultra-trace amounts, making them non-essential trace elements for humans [3].

Toxic trace elements are widely distributed in the environment, either from anthropogenic or natural sources [4]. Progressive industrialization and environmental degradation have contributed to increased human exposure to toxic trace elements today [5]. In fact, anthropogenic sources of toxic trace elements are notably larger than non-anthropogenic sources and are directly related to the polluted environment [6]. Accordingly, high levels of toxic trace elements can be found in the air, water, soil, plants, and animals [7]. Consequently, human biomonitoring studies have recorded differing levels of toxic trace elements in different biological materials among countries or even in different districts of the same country. In pregnant women, the main routes of exposure include dietary intake, contaminated drinking water, inhalation of polluted air, tobacco smoke, occupational exposure, and contact with contaminated environmental media [8]. Pregnancy is a physiologically altered state in females characterized by an increased need for essential nutrients [9]. However, it is also the most vulnerable developmental stage, as the intensive division and differentiation of fetal cells make the organism particularly susceptible to the association of environmental pollutants, including toxic trace elements [10]. In fact, toxic trace elements can impair women’s health even at low levels of exposure [11]. During pregnancy, toxic trace elements may cross the placental barrier, resulting in fetal exposure. As a result of maternal and placental transfer processes, exposure to toxic trace elements during pregnancy has consequences for the fetus’s prenatal health and the risk of numerous complications later in life, especially related to the nervous, immune, renal, and respiratory systems [12,13]. The impact of toxic trace elements on pregnancy has been assessed by their quantification in maternal blood, cord blood and/or placenta [14]. Maternal blood is collected in one or each of the three trimesters, usually in the last trimester or just before delivery [15]. Since the umbilical cord forms during the second and third trimesters, cord blood is a suitable biological material for assessing prenatal exposure to toxic trace elements because it reflects fetal blood at the time of birth following placental transfer during gestation [16]. The placenta is the largest fetal organ of limited duration (about 9 months) that plays a key role in protecting the fetus from multiple environmental pollutants [17].

Previous reviews and meta-analyses on this topic have yielded important findings [18,19,20]. However, these studies were primarily based on analysis of a single toxic trace element without a complete review of demographic and clinical pregnancy data. Also, data on toxic trace element levels in maternal and cord blood worldwide have not yet been consolidated. Thus, in this up-to-date narrative review, our primary objective was to summarize the literature data on As, Cd, Pb, and Hg levels in maternal and cord blood of healthy pregnant women across the world. Secondary objectives were to examine differences in the levels of these toxic trace elements between maternal and cord blood and to highlight the association between As, Cd, Pb, and Hg levels and demographic data, lifestyle, and pregnancy outcomes.

## 2. Materials and Methods

Study selection was performed in accordance with PRISMA 2020 guidelines. Titles were independently screened by two reviewers (RA and AS), followed by an independent full-text assessment for eligibility. Final screening of the literature was independently conducted by all reviewers. Disagreements were resolved through discussion and consensus among all reviewers, and when consensus could not be reached, a third reviewer made the final decision. All studies meeting predefined inclusion criteria were included in the review. No automation tools were used, and no additional information was sought from study investigators. The review protocol was not registered in a public database; however, all methodological steps, including search strategy and eligibility criteria, were predefined prior to data collection.

The primary aim of this review was to summarize concentrations of arsenic (As), cadmium (Cd), lead (Pb), and mercury (Hg) in maternal and cord blood, while reported associations with pregnancy outcomes were considered as secondary and were narratively described where available. Data extraction was independently performed and included study characteristics (design, sample size, and setting) and participant demographics (e.g., maternal age, smoking status, and residence). Missing or unclear data were recorded as not reported in the original studies, with no imputation performed. Due to substantial heterogeneity in study design, analytical methodologies, biological matrices, exposure assessment, and outcome definitions, the included studies were not sufficiently comparable to allow for meaningful quantitative synthesis. Therefore, the findings were synthesized narratively. No meta-analysis was conducted, as pooling of effect estimates would not have been methodologically appropriate due to the heterogeneity across studies and could potentially lead to misleading conclusions. Therefore, no summary effect estimates, statistical heterogeneity measures, or precision estimates were calculated. In line with the narrative design of the review, formal risk-of-bias assessment and certainty of evidence appraisal were not performed. Three major databases (PubMed, Cochrane Library, and Scopus) were used to search and select papers from 1 January 1990 to 14 May 2025. The time frame was chosen to ensure methodological comparability of analytical techniques used for trace element measurement across studies. The following keywords were used in the search: (“arsenic” or “cadmium” or “lead” or “mercury” or “methyl mercury” or “As” or “Cd” or Pb” or “Hg” or “heavy metal” or “toxic element”) AND (“maternal blood” or “umbilical cord blood” or “maternal serum/plasma” or “umbilical cord serum/plasma” pregnancy” or “trimester of pregnancy” or “demographics” or “lifestyle” or “tobacco smoking” or “pregnancy outcomes” or “postnatal anthropometry” or “preeclampsia” or “gestational diabetes” or “congenital heath defects” or “neural tube defects” or “pathological pregnancy”).

**Figure 1:**
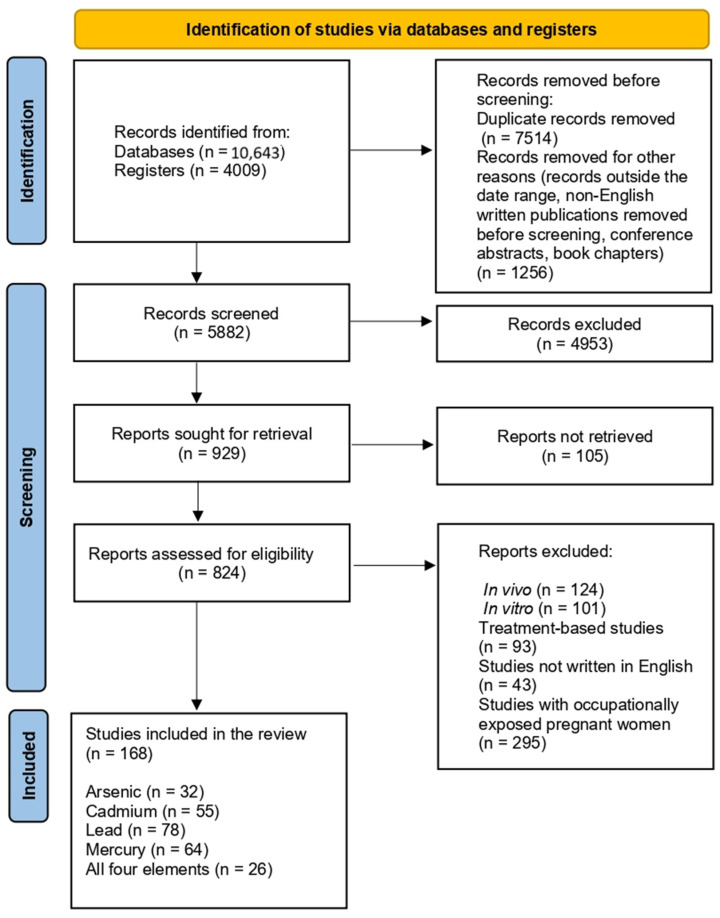
Details on the literature search, study identification, and inclusion/exclusion process for studies included in this review (PRISMA 2020 guidelines).

Details regarding the literature search and identification, screening, eligibility, inclusion and exclusion of studies are shown in Figure 1. Inclusion criteria were: (1) original full-length research papers (cross-sectional, prospective cohort, or case–control study design); (2) studies that measured As, Cd, Pb, and/or Hg in maternal blood and/or cord blood; (3) studies that examined toxic trace element levels in relation to demographics, lifestyle, and clinical outcomes of pregnancy. Exclusion criteria were: (1) in vitro, in vivo, and treatment-based studies; (2) studies without clearly stated pregnancy data (gestational age, comorbidities, period of blood sampling, the period during pregnancy when the blood was collected, etc.); (3) occupationally exposed pregnant women (unless otherwise stated in the text); (4) studies in which blood levels of toxic trace elements were determined by analytical techniques other than inductively coupled plasma mass spectrometry (ICP-MS), inductively coupled plasma optical emission spectrometry (ICP-OES), graphite furnace atomic absorption spectrometry (GF-AAS), flame atomic emission spectroscopy (FAAS), and cold vapor atomic absorption spectrometry (CV-AAS), while electrochemical and other methods/techniques were included only if the authors provided essential data, especially regarding the accuracy of elemental analysis; (5) studies with insufficient numerical data (box plots or other diagrams and images, without including numerical values in the text or tables); (6) studies reporting concentrations that were not comparable with the remaining literature due to apparent unit inconsistencies, reporting errors, or values several hundred- to thousand-fold outside the range observed in comparable populations were excluded; (7) studies not in English.

Studies could have assessed more than one toxic trace element and are therefore represented in multiple categories. As a result, category totals are not mutually exclusive and exceed the total number of included studies.

## 3. Results and Discussion

A total of 32 studies investigated arsenic (As) levels, including 20 studies reporting maternal blood concentrations and 24 studies reporting cord blood concentrations among healthy pregnant women worldwide. The largest number of studies was conducted in China (8 studies), followed by Spain in Europe (3 studies). Limited data were available from African countries, including Egypt and South Africa. For cadmium (Cd), 55 studies were included, with 38 reporting maternal blood levels and 45 reporting cord blood levels. The majority of studies originated from China (11 studies), with additional contributions from Scandinavian countries and Africa, while only three studies were conducted in the Americas.

Lead (Pb) was the most frequently studied element, with 78 studies identified across all inhabited continents. Of these, 50 studies reported maternal blood levels and 62 reported cord blood levels. For mercury (Hg), 64 studies were included, with 36 reporting maternal blood and 44 reporting cord blood concentrations. Most studies originated from China, followed by Spain and Canada, while data coverage across other European regions was limited. Twenty-six studies reported simultaneous analysis of all four elements (As, Cd, Pb, Hg). Regarding analytical methods, most studies used inductively coupled plasma mass spectrometry (ICP-MS) or graphite furnace atomic absorption spectrometry (GF-AAS) for As, Cd, and Pb, while cold vapor atomic absorption spectrometry (CV-AAS) was predominantly used for Hg.

The levels of all four elements were examined simultaneously in twenty-six studies.

According to Tables S1–S4, most of the studies used ICP-MS or GF-AAS techniques for total quantification of As, Cd, and Pb, while CV-AAS was primarily used to determine total Hg levels.

Median values presented in the figures were extracted as originally reported in the included studies, without recalculation or transformation of summary statistics.

### 3.1. Arsenic in Pregnancy

Arsenic is a non-essential trace element for pregnant women. Contaminated drinking water and food are the main sources of As in pregnancy [21]. The highest levels of As in groundwater were recorded in Bangladesh, India (West Bengal), Taiwan, Argentina, Chile, China, Mexico, the USA, Thailand, Hungary, Romania, Croatia, and Serbia [22,23]. Bangladesh in particular has a serious drinking water health crisis, with frequent cases of arsenicosis (As poisoning) [24,25]. Consequently, higher levels of As in pregnant women have been reported in Bangladesh and other As-contaminated countries than in areas with less As contamination. For example, Rahman et al. reported As levels of 11.9 μg/L in maternal blood and 15.7 μg/L in cord blood of pregnant women from Bangladesh [26]. In a study conducted in Argentina, where drinking water contained about 200 μg/L of As, its levels were 11.0 μg/L in maternal blood and 9.0 μg/L in cord blood [27]. These blood As levels were significantly higher than the numerical data shown in Table S1 for healthy pregnant women exposed to low levels of As, indicating that contaminated drinking water leads to increased As levels in maternal and cord blood.

High levels of exposure to As have been related to decreased birth weight, preterm births, fetal loss, and neonatal mortality [4,28]. In a meta-analysis conducted by Quansah et al., among populations with high exposure to As in drinking water (≥50 μg/L), As from groundwater was related to an increased risk of low birth weight, spontaneous abortion, stillbirth and to a moderately increased risk of neonatal and infant mortality [29]. However, the role of exposure to low levels of As during pregnancy is more challenging and will be discussed in more detail in this review.

#### 3.1.1. Maternal and Cord Blood as Levels Worldwide

**Figure 2:**
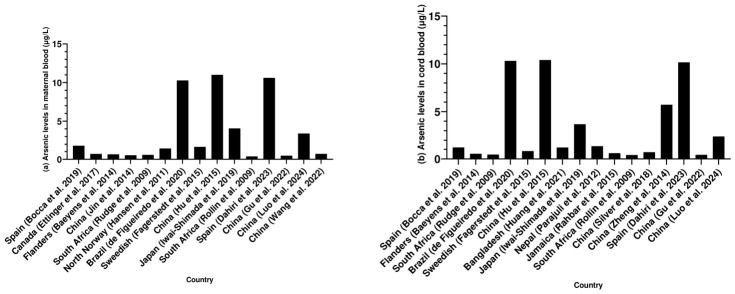
Median As levels in maternal blood (**a**) and cord blood (**b**) around the world [13,30,31,32,33,36,40,41,42,43,44,45,46,47,48,49,50,51,52].

Median levels of As in maternal and cord blood worldwide are shown in Figure 2. The lowest levels of As in maternal blood were found in South Africa (0.37 μg/L), but also in China, Belgium, and Canada (<1 μg/L) [30,31,32,33]. The highest levels of As in maternal blood were found in Egypt (59.7 ± 9.20 μg/L) [34]. The different maternal blood As levels in pregnant women from China could be explained by geographical differences, as there are regions in China with high natural concentrations of As in groundwater, but also regions with low concentrations [35].

The lowest levels of As in cord blood were found in two studies from South Africa (0.41 μg/L and 0.46 μg/L) and one study from China (0.43 μg/L), while the highest levels were found in Spain (20.3 ± 3.41 μg/L) [30,31,36,37]. Serbia ranked second among European countries with 17.2 ± 8.28 μg As/L in cord blood [38]. Regarding South America, we found only one study from Brazil, which reported a cord blood As level of 10.3 μg/L [39].

#### 3.1.2. Arsenic Levels During Pregnancy—Differences in as Levels Between Maternal and Cord Blood

According to the data in Table S1, only one study, conducted on 211 pregnant women in northern Norway, investigated As levels during pregnancy and from delivery to 6 weeks postpartum [40]. That study found As levels increased during this time. Therefore, further research is needed in this direction.

According to most of the studies presented in Table S1, As levels in maternal blood were significantly higher than in cord blood. Only two studies showed no statistically significant differences in As levels between maternal and cord blood. In most studies, positive and significant correlations in As levels between maternal and cord blood were found [41,42]. Bocca et al. emphasized the importance of the ratio of As in cord blood to that in maternal blood and reported a value of 0.70 for Tarragona, Spain [13,53]. We compared this ratio with those of other studies and found consistent ratios in South African and Flemish mothers (around 0.80 for both), but these ratios were lower than that in Bangladesh (1.30), noted for its areas with As contamination [32,36,53]. Although these observations may reflect differences in relative placental transfer or maternal–fetal exposure, they should be interpreted with caution, as maternal-to-cord blood ratios are influenced by multiple biological and methodological factors, and their biological and clinical significance has not yet been fully established.

#### 3.1.3. Arsenic Levels Depending on Demographics, Lifestyle, and Pregnancy Outcome

Regarding demographics and lifestyle, As levels in maternal and cord blood showed similar patterns, as levels in both blood matrices were higher in older pregnant women (versus younger pregnant women), non-smokers or former smokers (versus smokers), and in blood samples collected in spring (versus autumn or winter) (Table S1). Interestingly, Fagerstedt et al. reported that mothers who lived an anthroposophical lifestyle (a holistic lifestyle) had significantly higher blood As levels than mothers who did not live an anthroposophical lifestyle [43]. Although the number of studies examining the impact of As on pregnancy outcomes is insufficient, an interesting trend can be observed. Arsenic levels in maternal and/or cord blood have generally not been associated with adverse outcomes on preeclampsia, gestational diabetes, congenital heart defects, or anthropometric parameters. The correlation of maternal blood As level with gestational age was inversely proportional, which is also consistent with data on high exposure to As from the environment [54]. On the other hand, the correlation of As from cord blood with gestational age was inconsistent across studies and exposure settings. For example, Cabrera-Rodríguez et al. did not find significant differences in cord blood As levels with SGA (small for gestational age), AGA (appropriate for gestational age), and LGA (large for gestational age), while Huang et al. (2021) [23] found a significant association of As in cord blood with SGA. In fact, they pointed out that As in cord blood was the most important predictor of SGA. However, compared to the study by Cabrera-Rodríguez et al. (2018) [55]., which was conducted in Spain, the study by Huang et al. (2021) [23]. was conducted in Bangladesh. We stress that the findings of Huang et al. (2021) could reflect environmental pollution in Bangladesh, as previously explained.

In summary, our observations regarding As are as follows: data on As levels by trimester of pregnancy are lacking, demographic findings on blood As levels are fairly uniform, and low As levels do not play a significant role in the clinical course of pregnancy versus high As levels, but could be related to gestational age. We emphasize that further research is needed to examine the association of low levels of As with pregnancy outcomes. Moreover, since As is present in the body in numerous inorganic and organic forms (As3+, As5+, monomethylarsonic acid, dimethylarsinic acid, arsenosugars, arsenolipids, etc.), it is necessary to conduct As speciation in the future and thus qualitatively and quantitatively detect As-species of clinical/toxicological interest.

### 3.2. Cadmium in Pregnancy

Cadmium is a non-essential metal with a very long half-life in the body [56]. Cadmium (Cd) exposure during pregnancy has been investigated in a number of biomonitoring studies, although reported findings on pregnancy outcomes remain heterogeneous. Overall, higher Cd levels have been associated in some studies with adverse pregnancy outcomes, including reduced birth weight, preterm birth, and altered neonatal outcomes, although results are not consistent across populations. The observed differences in findings may be related to variations in exposure levels, population characteristics, and study design, including differences in adjustment for key confounders such as nutritional status and co-exposure to other trace elements. At lower exposure levels, the evidence for adverse pregnancy effects is less consistent, and several studies have reported weak or non-significant associations with pregnancy outcomes [12,57,58,59,60,61].

#### 3.2.1. Maternal and Cord Blood Cd Levels Worldwide

**Figure 3:**
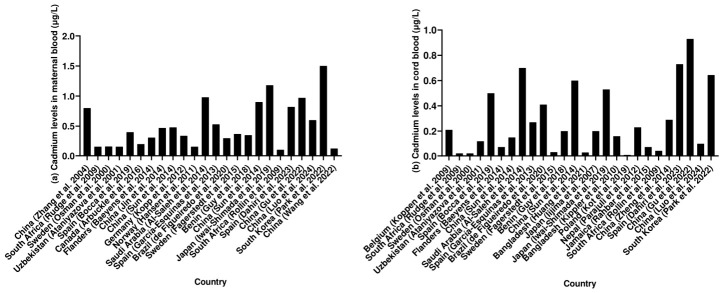
Median Cd levels in maternal blood (**a**) and cord blood (**b**) around the world [12,13,30,31,32,36,40,42,43,44,46,47,48,49,50,52,61,64,65,66,67,68,69,70,71,72,73,74,75].

Median levels of Cd in maternal and cord blood worldwide are shown in Figure 3. The highest level of Cd in maternal blood was observed in a study from Turkey (46 ± 90 μg/L) [62]. The higher presence of Cd in Turkey has been well documented in various areas due to industrial pollution, the use of artificial fertilizers, and the high Cd levels in fish and shellfish harvested from the Black Sea [63]. The lowest maternal Cd levels were found in studies from South Africa (0.02 μg/L) and Sweden (0.02 μg/L) [36,64]. In other studies we analyzed from around the world, Cd levels in maternal blood were generally low, around 1 μg/L (Table S3).

Regarding cord blood Cd levels worldwide, we noted that the previously mentioned study from Turkey also reported the highest cord blood Cd levels (22 ± 22 μg/L) [62]. Finally, we can notice that, apart from the highest values already described, there are no significant deviations in cord blood Cd levels between different parts of the world.

#### 3.2.2. Cadmium Levels During PregnancyDifferences in Cd Levels Between Maternal and Cord Blood

Most studies agreed that Cd levels in maternal blood were higher at the end than at the beginning of pregnancy (Table S2). Yet, data on Cd levels by trimester of pregnancy are lacking, so new research in this direction is needed.

According to all studies shown in Table S2, Cd levels in maternal blood were significantly higher than in cord blood, with (*p* < 0.05) or without (*p* > 0.05) a significant positive correlation between the two blood compartments. These findings indicate that, although Cd can cross the placenta, its transplacental transfer is limited, as reflected by consistently lower Cd concentrations in cord blood than in maternal blood. In fact, metallothionein (MT) expression is thought to regulate the limited transport of Cd across the placenta [44,76]. Cadmium induces the expression of MTs, which are synthesized in certain maternal organs and the placenta itself estimated that the transplacental transport of Cd is low (about 8%), which is in agreement with Kopp et al. (2012) and Osman et al. (2000) [43,61,64,65]. Interestingly, Ronco et al. (2005) showed that tobacco smoking increases MT synthesis due to increased Cd accumulation in the placenta; in this organ, they found 0.075 µg Cd/g dry weight in smokers compared to 0.024 µgCd/g dry weight in non-smokers [77].

#### 3.2.3. Cadmium Levels Depending on Demographics, Lifestyle, and Pregnancy Outcome

One cigarette contains 0.5–2.0 µg of Cd; thus, one cigarette raises blood Cd levels by 0.1–0.2 µg/L [34,78]. Smokers usually have approximately three times higher blood levels of Cd than non-smokers [79]. According to Table S2, studies have consistently shown that passive inhalation of tobacco smoke and active tobacco smoking increase maternal blood Cd levels compared to pregnant women who do not consume tobacco. For example, Pizent et al. found that mothers who smoked during pregnancy had 0.68 µg/L of Cd in their blood, about double that of mothers who did not smoke during pregnancy (0.31 µg/L) [80]. However, they found no significant difference in cord blood Cd levels (0.029 µg/L for smokers vs. 0.028 µg/L for nonsmokers). Osman et al. (2000) noted that the median weight of infants born to smoking mothers was approximately 0.2 kg less than that of non-smoking mothers [64]. Baeyens et al. (2014) [32] reported that maternal blood Cd levels increased with the number of cigarettes smoked. On the other hand, studies showed that Cd levels did not change in cord blood depending on maternal tobacco consumption, further confirming limited transplacental transfer of Cd (details in Table S2). 

According to the literature data presented in Table S2, Cd levels in maternal and/or cord blood were not significantly associated with preeclampsia, preterm birth, women with preterm premature rupture of membranes (PPROM) compared to women without PPROM, or congenital heart defects. However, the number of studies is small, so new ones are needed. The effect of Cd on Apgar score and gestational age appears to be negatively associated, especially with SGA. On the other hand, the impact of Cd on anthropometric parameters is controversial. Lee et al. (2021) showed that higher cord blood Cd levels were negatively associated with mean birth weight and lower mean head circumference after adjusting for numerous covariates, while Tang et al. (2016) showed no significant associations between Cd exposure and birth weight, height, and head circumference after adjusting for numerous covariates [78,81]. Recently, Zinia et al. (2023) showed that maternal blood Cd levels during early pregnancy and late pregnancy were significantly associated with birth weight (i.e., high levels were associated with low birth weight) [82]. These discrepancies between studies need to be further elucidated, as birth weight is one of the main indicators/predictors of a healthy pregnancy outcome [45,83].

In summary, our observations regarding Cd are as follows: data on Cd levels by trimester of pregnancy are lacking, it appears to be an important contributor to elevated blood Cd in pregnancy, and the association of environmental Cd levels with the clinical course of pregnancy needs to be studied in the context of smokers versus non-smokers. In addition to Cd levels in maternal and cord blood, researchers should also focus on quantifying MTs. Thus, further research is urgently needed to answer these key questions.

### 3.3. Lead in Pregnancy

According to the ATSDR, Pb is listed as the second most toxic substance. According to the WHO, Pb toxicity has been associated with an estimated 600,000 new cases of intellectual disability in children each year, and as many as 99% of children affected by Pb burden live in developing countries [84]. The half-life of Pb in the bloodstream is about 30 days [84]. At high levels, Pb exposure has been associated with reduced birth weight and length, congenital malformations, spontaneous abortions, preeclampsia, and impaired neurodevelopment [44,61]. Edwards showed that higher levels of Pb in water were correlated with higher rates of fetal mortality during the “lead crisis” in the USA (2000–2004) [85,86]. However, Pb also crosses the blood–brain barrier; in fact, Pb has been detected in the fetal brain at 13 weeks of gestation [84]. The previously proposed “safe” blood Pb level of 100 µg/L prescribed by the Centers for Disease Control and Prevention (CDC) has been reduced to 50 µg/L [12]. The reason was that studies had reported that blood Pb levels < 100 µg/L were associated with numerous prenatal and postnatal complications, as well as complications later in life, especially up to the age of 6, but also later in life [87,88]. According to a study by Gilbert and Weiss about 20 years ago, there was sufficient scientific evidence to reduce blood Pb levels to 20 µg/L to prevent the negative neurobehavioral effects of Pb [89]. The European Food Safety Authority (EFSA) has determined that an increase in blood Pb of 12 µg/L reduces IQ by one unit [66]. Moreover, according to most authors, including us, there is no evidence suggesting a safe threshold of blood Pb levels in the blood of pregnant women, children, and the general population [90,91].

#### 3.3.1. Maternal and Cord Blood Pb Levels Worldwide

**Figure 4:**
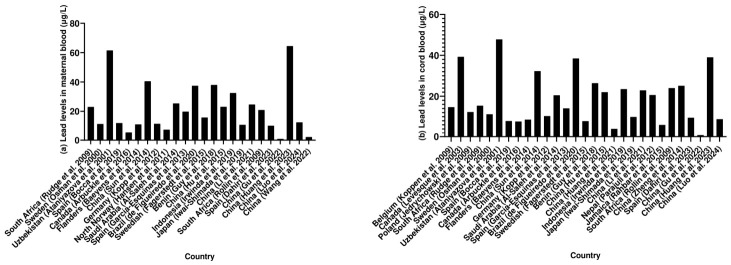
Median Pb levels in maternal blood (**a**) and cord blood (**b**) around the world [13,30,31,32,36,40,42,43,44,45,46,48,49,50,52,61,64,65,66,67,68,71,74,94,95,96,97,98,99].

Median levels of Pb in maternal and cord blood worldwide are shown in Figure 4. The lowest Pb levels were found in France, both in maternal blood (0.18 ± 0.12 µg/L) and in cord blood (0.15 ± 0.03 µg/L) [92]. The highest levels of Pb in maternal blood were found in Turkey (154 ± 98.5 µg/L), followed by China (130 ± 122 µg/L) [41,62]. The same study from Turkey that reported the highest maternal blood Pb levels also reported the highest cord blood Pb levels (151 ± 123 µg/L) [62].

France is known as one of the countries with the lowest levels of Pb in the blood of adults and children. The reasons are most likely related to the very early phase-out of leaded gasoline, low industrial and mining risks, and regulated imports of food and cosmetics, which further reduce the potential Pb intake of pregnant women [93]. High blood Pb levels in Turkey and China can be attributed to a combination of industrial, environmental, and regulatory factors. However, one study from China showed significantly lower Pb levels in maternal (0.87 µg/L) and cord blood (0.78 µg/L) [31]. This was the only study in which Pb levels reported in maternal blood and cord blood differed from other studies in China, wherein the Pb levels in both blood compartments were generally higher, as already mentioned. For example, Li et al. reported higher values in China, with maternal blood lead levels of 23.1 ± 21.2 μg/L, while the levels in umbilical cord blood were 14.2 ± 7.6 μg/L [94].

#### 3.3.2. Lead Levels During Pregnancy—Differences in Pb Levels Between Maternal and Cord Blood

Lead levels during pregnancy differ notably depending on the degree of maternal exposure to this toxic metal. However, blood Pb levels during pregnancy were usually lower compared to blood Pb in women who were not pregnant or after giving birth (Table S3).

According to the data in Table S3, Pb levels in maternal blood were always higher than in cord blood and were positively correlated, with (*p* < 0.05) or without (*p* > 0.05) a statistically significant difference.

#### 3.3.3. Lead Levels Depending on Demographics, Lifestyle, and Pregnancy Outcome

Studies agree that environmental Pb levels have decreased after the removal of tetraethyl Pb (an antiknock agent) from gasoline [100]. Perhaps the best example is the study by Levin et al. (2021) [101]. which compared Pb levels in maternal and cord blood in age-matched women from Paris (France, *n* = 206) and Montreal (Canada, *n* = 160), since at the time of the completed sampling period (1992–1995), France was still using tetraethyl Pb, while Canada was no longer using it (they had replaced tetraethyl Pb with methylcyclopentadienyl manganese tricarbonyl). They found significantly higher levels of Pb in the blood of women from Paris than in women from Montreal (54.0 ± 14.0 µg/L versus 21.0 ± 17.0 µg/L), and in cord blood from Paris (32.0 ± 20.0 µg/L) than in cord blood from Montreal (17.0 ± 17.0 µg/L) [102]. However, higher Pb levels have still been recently recorded in urban areas compared to rural areas worldwide, so pregnant women in urban areas could also have higher blood Pb levels than those with rural lifestyles [38].

According to Table S3, blood Pb levels do not usually show a relationship with maternal age, but can be significantly higher in pregnant women over 35 years of age. Also, studies show that household paints and kohl (also called surma, an eye cosmetic) are still sources of Pb for pregnant women. Tobacco use increases Pb levels in maternal blood and cord blood, but not to the same extent as it elevates Cd levels. Numerous studies have highlighted that high levels of Fe (hemoglobin), vitamin D, Ca, and Zn could have a significant impact on reducing circulating Pb levels (Table S3). According to Ettinger et al. (2007) [33], Ca supplementation during pregnancy should be supported, as increasing Ca levels reduces Pb levels in the bloodstream of both the mother and the fetus or infant, later in life. Some amount of Pb in a mother’s bloodstream could be of endogenous rather than exogenous origin [103]. Thus, during pregnancy, Al-Saleh et al. (2011) [12] pointed out that Pb can be released from the skeletal system through demineralization, as bones are one of the main reservoirs of Pb, which is estimated to increase the level of Pb in the mother’s blood by 15–20%. In the presence of sufficient Ca, this effect was minimized [12]. At the molecular level, studies have shown that the presence of low levels of Pb disrupts Ca transport in syncytiotrophoblasts, as well as leading to the deposition of Pb-Ca complexes in the microvilli around the trophoblast (placental calcification) and other events [104,105]. In a randomized placebo-controlled trial in Mexico City, where blood Pb levels were around 40 µg/L, CaCO_3_ supplementation of 1.2 g daily was related to a modest reduction in blood Pb levels when administered during pregnancy [106].

According to Table S3, several studies indicated that Pb levels in maternal or cord blood were associated with neonatal disorders, inadequate anthropometric parameters (especially head circumference), poor cognitive development of the child, and mental disorders, while they were not significantly associated with premature birth. However, the direction and strength of these associations varied across studies, and findings were not entirely consistent, so more studies are urgently needed to confirm or refute these conclusions.

In summary, our observations regarding Pb are as follows: data on Pb levels by trimester of pregnancy are lacking, there is a significant negative association between low blood Pb with pregnancy outcomes, anthropometric indicators and later child development, while randomized clinical controlled double-blind studies are needed to determine at what levels the Pb in maternal blood and cord blood begin to decline after administration of Fe, Zn, and Ca. It is also necessary to further investigate the association of low levels of Pb in maternal blood and cord blood with pathological pregnancy outcomes.

### 3.4. Mercury in Pregnancy

According to the ATSDR, Hg is listed as the third most toxic substance [57]. In addition to natural sources, coal-fired power plants are among the major anthropogenic sources of environmental Hg, which is converted by microorganisms into methylmercury (MeHg) and bioaccumulates in aquatic food chains [107,108,109]. The transfer of Hg from mother to fetus depends on its chemical form [110]. Inorganic Hg crosses the placenta inefficiently, whereas organic forms, particularly MeHg, readily reach the fetus and may adversely affect neurodevelopment [46,111,112,113]. Elevated cord blood Hg levels have been associated with impaired neurocognitive and psychomotor development, deficits in language, memory, and attention, and reduced IQ [95,114,115]. Consequently, the US EPA and FDA have established exposure recommendations for pregnant women and advised limiting consumption of fish species with high Hg concentrations [13,116]. The severe consequences of prenatal MeHg exposure were demonstrated by the well-documented poisoning episodes in Minamata and Niigata, Japan, and in Iraq [109,117]. Mercury may also induce metallothionein expression and interact with essential elements such as Zn, Se, and Fe, thereby influencing its toxicity [118,119].

#### 3.4.1. Maternal and Cord Blood Hg Levels Worldwide

**Figure 5:**
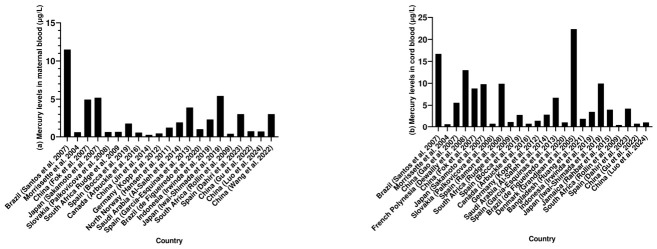
Median Hg levels in maternal blood (**a**) and cord blood (**b**) around the world [13,16,30,31,36,40,42,44,46,47,48,49,61,65,67,96,98,109,126,127,128,129,130,131,132].

Median levels of Hg in maternal and cord blood worldwide are shown in Figure 5. The highest Hg levels in maternal blood were found in Egypt (28.8 ± 11.8 µg/L) and Turkey (24.0 ± 7.13 µg/L) [34,120]. The lowest Hg levels in maternal blood were reported in a study from China (0.26 µg/L) [47]. In cord blood, the highest Hg levels were found in Greenland (35.6 ± 32.0 µg/L) and the Faroe Islands (22.4 µg/L), while the lowest Hg levels were recorded in Turkey (0.50 µg/L) [16,121,122]. In Greenland, although geographically distant from major industrial zones, pollutants can be transported by air currents/wind. In fact, Hg is often transported from industrial areas into the atmosphere and can even end up in Greenland, where it accumulates in water and soil [123]. Additionally, Greenlanders eat a lot of seafood, including top-of-the-range marine mammals [124]. In Denmark, multiple industrial and agricultural activities could contribute to higher levels of Hg in the environment and consequently its intake in pregnant women [125].

#### 3.4.2. Mercury Levels During Pregnancy—Differences in Hg Levels Between Maternal and Cord Blood

Mercury levels during pregnancy differ significantly depending on the degree of maternal exposure to this toxic trace metal. However, blood Hg levels during pregnancy were usually lower compared to blood Hg in women who were not pregnant or after giving birth (Table S4).

According to Table S4, Hg and MeHg levels in cord blood were at least twice as high as maternal blood levels. Mercury levels between the two blood compartments were positively correlated, usually with statistical significance (*p* < 0.05). This finding likely reflects efficient placental transfer of Hg and its preferential binding within the fetal circulation, resulting in cord blood Hg concentrations that may exceed those observed in maternal blood. This can be explained by the stronger binding of Hg to hemoglobin in fetal erythrocytes [34]. Namely, fetal hemoglobin and hematocrit levels were higher in pregnant women than in adult non-pregnant women [133,134]. In addition to hemoglobin, α-fetoprotein, a specific protein of fetal serum albumin, has a higher affinity for Hg in cord blood than Hg in maternal blood [12].

#### 3.4.3. Mercury Levels Depending on Demographics, Lifestyle and Pregnancy Outcome

As expected, maternal and fetal blood Hg levels were higher in pregnant women living near seashores (Mediterranean, Arctic, Japan, Korea, and others). This claim is also supported by a meta-analysis that included 10 studies conducted in countries where people frequently consume fish and seafood and found that blood MeHg levels were approximately 1.7 times higher in newborns compared to their mothers [135]. Also, Jin et al. noted that women who consumed fish three or more times a week had blood Hg levels that were about 35% higher than women who consumed fish one to two times a week, who in turn showed Hg levels that were 29% higher than women who consumed fish once a week [47]. However, there is still no clear consensus on the benefits/risks of consuming fish and seafood regarding MeHg and essential nutrients (polyunsaturated fatty acids, omega-3 and -6 fatty acids, vitamin D, selenium (Se), etc. According to Golding et al., blood Hg levels were also associated with the number of maternal amalgam fillings, and blood Hg levels in smokers, former smokers, and nonsmokers did not differ significantly (Table S4) [136].

Although literature findings on pregnancy outcomes were inconsistent, we observed the following trends: (1) maternal blood Hg levels were generally negatively associated with birth weight, (2) cord blood Hg levels were generally not significantly associated with gestational age or anthropometric parameters. As an explanation for these findings, we would point to the potential protective effect of Se, vitamin E, and docosahexaenoic acid (DHA) consumed by pregnant women via food or supplements. However, Wang et al. reported that each unit (µg/L) of Hg blood plasma level was related to a 2.88-fold increased risk of congenital heart defects after adjustment. Thus, further research is needed in this direction [48].

In summary, our observations regarding Hg are as follows: data on Hg levels by trimester of pregnancy are lacking, demographic findings on circulating Hg levels are fairly consistent and associated with (shell) fish consumption, and high environmental Hg levels have been associated with adverse neurological outcomes in children, but these can be minimized or even prevented. With the support of the American College of Obstetricians and Gynecologists, the FDA and EPA recommend that pregnant and breastfeeding women eat 2–3 servings of low-Hg fish per week for nutritional benefits such as omega-3 fatty acids, while avoiding high-Hg species to balance fetal brain development with the risk of MeHg exposure. However, the latter statement needs to be substantiated, ideally by clinically controlled double-blind studies. Similar to As, Hg speciation is necessary for reliable assessment of inorganic Hg and organic Hg (particularly MeHg) levels in maternal and cord blood in relation to pregnancy outcome.

Potential confounding factors, including dietary intake, socioeconomic status, lifestyle habits, and co-exposure to other environmental pollutants, may also influence maternal blood metal levels and pregnancy outcomes. These factors were not consistently controlled for across the included studies, which may affect the interpretation and comparability of the reported findings.

A limitation of this review is the heterogeneity in analytical methodologies applied across the included studies, including differences in techniques for metal determination (e.g., ICP-MS, AAS), biological matrices analyzed (whole blood, serum, plasma), detection limits, and laboratory protocols. Such variability may influence the comparability of reported concentrations and should be considered when interpreting and synthesizing the evidence.

## 4. Conclusions

In summary, toxic trace element levels in maternal and cord blood appear to vary across different populations and geographic regions, which can be linked to the environment and the degree of maternal exposure to As, Cd, Pb, and Hg. However, due to the heterogeneity of the available evidence, no unexpected or clearly systematic global differences can be confirmed. Regarding the differences in toxic trace element levels between maternal and cord blood, we observed differences; As and Cd levels were reported in the included studies to be higher in maternal blood than in cord blood, while Pb and Hg (including MeHg) levels were reported in the included studies to be higher in cord blood than in maternal blood. For each of the four elements, positive correlations between maternal and cord blood concentrations were reported across studies, with statistically significant associations observed in some studies (*p* < 0.05), while others reported non-significant correlations (*p* > 0.05). Low circulating As levels were not notably associated with pathological pregnancy outcomes or anthropometry. Tobacco smoking was frequently reported as an important source of Cd in pregnancy; smokers had significantly higher levels of Cd in maternal blood (but not necessarily in cord blood) than nonsmokers. Some studies, mainly in low-exposure populations, reported that low levels of circulating Cd were negatively associated with gestational age and Apgar score, although these findings were not consistent across all studies. Low levels of circulating Pb were reported in several studies to be associated with preeclampsia, congenital heart defects, undesirable anthropometric changes (especially in head circumference), and neuropsychiatric impairment later in life. However, these associations were more frequently reported in high-exposure settings and in some adjusted analyses, while unadjusted and low-exposure studies showed less consistent results. Methyl mercury is the major form of Hg in pregnancy, and demographic data indicate higher levels of MeHg in the blood of pregnant women who live near seashores than in the blood of those who do not. Although Hg and MeHghave been associated with adverse pregnancy outcomes, their effects seem to be stronger after birth, particularly on neurological damage later in life. However, with adequate amounts of minerals and vitamins, the adverse effects of Hg (but also Pb) on pregnancy may be reduced. Overall, we can conclude that the intrauterine environment of the fetus is not completely protected from the harmful effects of toxic trace elements from the environment, placental transfer varies depending on the element and chemical form, and all four trace elements (As, Cd, Pb, and Hg) may cross the placenta to different extents, as reflected in cord blood levels reported in the included studies. Consequently, Pb shows relatively consistent evidence of adverse effects in the included studies, particularly in the domain of neurodevelopment. Furthermore, since there are no safe levels of toxic trace elements for transplacental transfer, it is necessary to implement new strategies to protect pregnant women from exposure to them. Some of these would include educating pregnant women about the ways elements enter the body, quitting smoking, avoiding leaded tap water, using indoor air purifiers, and stopping eating seafood and fish contaminated with high levels of MeHg. On a global scale, it is necessary to reduce metal emissions. Overall, monitoring of toxic trace element levels in the blood during pregnancy and early childhood should be supported.

### Further Perspectives

We can summarize that much has been done in the field of examining the total level of As, Cd, Pb, and Hg in the blood of healthy pregnant women. The future research directions can be structured into several key areas. First, regarding trimester-specific sampling, while many studies have measured total levels of As, Cd, Pb, and Hg in maternal blood, there is still a lack of clear evidence on how these levels change across pregnancy. This highlights the need for standardized and trimester-specific sampling protocols in future studies. Second, in terms of exposure-source characterization, future studies should better integrate toxic trace element measurements with detailed information on environmental, dietary, occupational, and sociodemographic sources of exposure in order to improve the interpretation of observed levels. Third, regarding maternal–cord blood relationships, more research is needed to better quantify and standardize maternal-to-cord blood ratios, as they may provide complementary information on maternal–fetal exposure. However, these ratios are currently inconsistently reported in the literature, and their biological and clinical significance remains uncertain. Fourth, concerning pregnancy outcomes, future studies should focus on better-controlled designs that assess the association between toxic trace element exposure and both healthy and pathological pregnancy outcomes, ideally with adjustment for key confounders. Although our goal was not to include data on element speciation, it is undoubtedly pivotal in the future to examine the impact of species of all four trace elements, especially As and Hg, on both healthy and pathologically altered pregnancies.

## Data Availability

No new data were created or analyzed in this study. Data sharing is not applicable to this article.

## Supplementary Materials

The following supporting information can be downloaded at: https://www.mdpi.com/article/10.3390/jox16040132/s1, Table S1: Arsenic (As) levels in maternal and cord blood of non-occupationally exposed healthy pregnant women worldwide (µg/L).; Table S2: Cadmium (Cd) levels in maternal and cord blood of non-occupationally exposed healthy pregnant women worldwide (µg/L); Table S3: Lead (Pb) levels in maternal and cord blood of non-occupationally exposed healthy pregnant women worldwide (µg/L); Table S4: Mercury (Hg) levels in maternal and cord blood of non-occupationally exposed healthy pregnant women worldwide (µg/L). The references [137,138,139,140,141,142,143,144,145,146,147,148,149,150,151,152,153,154,155,156,157,158,159,160,161,162,163,164,165,166,167,168] are cited in the supplementary materials.
