# Supplementary material for: Toxic Trace Element Levels in Maternal and Cord Blood Worldwide and Their Impact on Pregnancy: A Narrative Review
Source: J Xenobiot. 2026 Jul 14;16(4):132. doi: 10.3390/jox16040132 (PMC13398213; doi:10.3390/jox16040132)
Supplement: Supplementary file 1 [file jox-16-00132-s001.zip › jox-4367903-supplementary.pdf]

Supplementary Materials: Toxic Trace Element Levels in Maternal and Cord Blood Worldwide and Their Impact on Pregnancy: A Narrative Review

Radomir Aničić, Dejan Mihajlović, Jovana Kocić and Aleksandar Stojsavljević

Table S1. Arsenic (As) levels in maternal and cord blood of non-occupationally exposed healthy pregnant women worldwide (μg/L).

**Reference**	**Country**	***N***	**Blood collection time period**	**Biological material**	**Analytical technique**	**As levels (μg/L)**		**Main findings**
						Maternal	Cord	
Abdel Hameed et al., 2019 [34]	Cairo, Egypt	113	2016-2017	Serum	ICP-MS	59.7 ± 9.20	3.50 ± 7.60	Significant negative association between maternal serum As level and gestational age. Significant negative correlations between maternal serum As level and both 1-minute and 5-minute Apgar scores.
Bermúdez et al., 2015 [37]	Valencia, Spain	54	NP	Plasma	ICP-MS	2.01 ± 1.24	20.3 ± 3.41	No relationship between anthropometric parameters and As levels in either the maternal or cord plasma.
Bocca et al., 2019 [13]	Tarragona, Spain	53	2016-2017	Blood	SF-ICP-MS	1.80 (0.30-7.30)	1.20 (0.3-5.9	Significantly lower As levels in cord blood than in maternal blood at delivery, suggesting that the placenta could modulate As transfer.

Ettinger et al., 2017 [33]	10 cities, Canad a	2001	2008-2011	Blood	ICP-MS	0.69	-	Significantly higher As levels in maternal blood were found in women who were older, foreign-born (predominantly from Asian countries), former or never smokers, nulliparous, with a university education,

								and whose blood sample was taken in the spring (compared to the fall).
Baeyens et al., 2014 [32]	Flanders	255	2007-2011	Blood	HR-ICP-MS	0.64	0.54	Strong positive correlation between As levels in maternal and cord blood, indicating that As is transported across the placenta. Significant positive correlation between cord blood As level and maternal age. Negative association of maternal blood As level and smoking during pregnancy. Statistically lower As levels in maternal blood and cord blood in mothers who smoked during pregnancy than in controls.
Jin et al., 2014 [47]	China	215	2005-2009	Blood	ICP-MS	0.52	-	Maternal blood As level was significantly higher in Yushe County than in Daixian County, which had a higher prevalence of NTDs. Association of higher maternal blood As levels with consumption of fish, shrimp, or shellfish, as well as consumption of beef, pork, or mutton.
Rudge et al., 2009 [36]	South Africa	62	NP	Blood	ICP-MS	0.57	0.46	Arsenic crossed the placental barrier by diffusion (approximately 80%).

Hansen et al., 2011 [40]	North Norway	211	2007-2009	Blood	ICP-MS	1.40	-	Arsenic levels in the blood increased during pregnancy and from birth to 6 weeks postpartum. Consumption of fish, especially

								shellfish, was a strong positive predictor of higher blood levels As.
Cabrera-Rodríguez et al., 2018 [55]	Canary Islands, Spain	471	2015-2016	Blood	ICP-MS	-	1.36 ± 3.02	No significant differences between cord blood As levels with SGA (n = 47), AGA (n = 377), and LGA (n = 47).
de Assis Araujoal. , 2020 [39]	Rio de Janeiro, Brazil	140	2017-2018	Blood	ICP-MS	10.27	10.31	Significant positive correlation between As levels in maternal and cord blood.
Fagerstedt et al., 2015 [43]	Stockholm, Swedish	330	2004-2007	Erythrocytes	ICP-MS	1.60	0.82	Significantly higher maternal erythrocyte As levels in non-anthroposophical mothers than in mothers with an anthroposophical lifestyle.
Hu et al. (2015) [45]	Four cities (Beijing, Lanzhou, Taiyuan, and Xiamen), China	81	2011	Blood	ICP-MS	11.0	10.4	Significant difference between As levels in maternal blood and cord blood. No significant associations between As levels in maternal blood or cord blood and birth weight.
Huang et al., 2021 [23]	Sirajikhan and Pabna	745	2008-2011	Serum	ICP-MS	-	1.19 (0.72–2.46)	Arsenic was the most important predictor of SGA. Significantly higher cord serum As levels in premature babies than in

								term

	Sadar Upazila, Bangladesh							babies. Significant association of cord blood As level with risk of preterm birth.
Iwai-Shimada et al., 2019 [46]	Tohoku region, Japan	687	2001-2006	Blood	ICP-MS	4.06 (2.68-6.81)	3.68 (2.58-5.25)	Significantly lower As level in cord blood than in maternal blood.
Lee et al., 2020 [78]	Sirajdikhan and Pabna Sadar Upazilas, Bangladesh	1088	2008-2011	Blood	ICP-MS	-	8.80 ± 11.3	No significant association between cord blood As level and birth weight.
McKeating et al., 2020 [157]	Queensland, Australia	2879	2006-2010	Blood	ICP-MS	-	0.9 ± 1.83	No significant differences between cord blood As levels in control group and pathological groups (hypertensive, PE, GDM, IUGR, preterm, and/or post-term delivery).
Parajuli et al., 2012 [50]	Terai region, Nepal	100	2008	Blood	ICP-MS	-	1.33 (0.51-9.58)	Significantly higher cord blood As levels in less educated mothers than in educated mothers. Negative correlation between cord blood As level and maternal age.
Rahbar et al., 2015 [44]	Kingston, Jamaica	100	2011	Blood	ICP-MS	-	0.60	No significant association of cord blood As level and head circumference.

Rollin et al., 2009 [30]	Seven geographical areas, South Africa	96	2005-2007	Blood	ICP-MS	0.37 (0.08-0.67)	0.41 (0.09-1.26)	Significant correlation between As levels in maternal and cord blood. No significant differences between As levels in maternal blood or cord blood As levels in the seven geographical areas.
Sakamoto et al., 2010 [115]	Munakata City, Fukuoka, Japan	81	NP	Erythrocytes	ICP-MS	6.16 ± 3.40	3.76 ± 2.27	Significantly lower As levels in cord erythrocytes than in maternal erythrocytes. Significant correlation between As levels in maternal and cord blood.
Silver et al., 2018 [51]	Zhejiang Province, China	357	2008-2011	Blood	ICP-MS	-	0.70	Infants born in spring had approximately 0.1-0.2 μg/L higher cord blood As levels and more than twice the likelihood of cord As than infants born in autumn/winter.
Stojsavljević et al., 2022 [38]	Serbia	125	2020-2021	Plasma	ICP-MS	34.8 ± 7.38	17.2 ± 8.28	Significantly higher As levels in maternal blood than in cord blood.
Zheng et al., 2014 [52]	Xiamen, China	1106	2010	Serum	ICP-MS	-	5.72 (3.39-9.33)	No significant difference between cord As levels in the group with adverse pregnancy outcomes and the control group.
Zhou et al., 2019 [41]	Beijing, China	52	2013-2014	Blood clots	ICP-MS/MS	8.34 ± 5.72	7.38 ± 5.58	No significant difference between As levels in maternal and cord blood clots.

Dahiri et al., 2023 [42]	Seville, Spain	100	2020-2021	Blood	ICP-MS	10.6	10.2	No significant difference between As levels in maternal blood and cord blood.

Gu et al., 2022 [31]	Peking, China	48	NP	Plasma	ICP-MS	0.46 (0.25-1.04)	0.43 (0.26-0.76)	No significant difference between As levels in maternal blood and cord blood. Significant correlation between As levels in paired maternal and cord plasma.
Luo et al., 2024 [49]	Zhejiang province, China	48	2018-2019	Blood	ICP-MS	3.40 (2.20-5.00)	2.40 (1.60-3.70)	Positive correlation between As levels in cord and maternal blood.
Wang et al., 2022 [48]	Eight provinces of China	303 CHD cases and 303 healthy controls	2016-2020	Plasma	ICP-MS	0.70 (0.41-1.19)	-	No differences in plasma As levels between cases with CHD and controls. No association between As levels and CHD risk.

Abbreviations: *N* – total number of participants; NP – not presented; ICP-MS – inductively coupled plasma mass spectrometry; SF – sector field; HR – high resolution; MS/MS – tandem mass spectrometry; NDTs – neural tube defects; SGA – small for gestational age; AGA – appropriate for gestational age; LGA – large for gestational age; PE – preeclampsia; GDM – gestational diabetes mellitus; IUGR – intrauterine growth restriction; CHDs – congenital heart defects.

Table S2. Cadmium (Cd) levels in maternal and cord blood of non-occupationally exposed healthy pregnant women worldwide (μg/L).

**Reference**	**Country**	***N***	**Blood collection time period**	**Biological material**	**Analytical technique**	**Cd levels (μg/L)**		**Main findings**
						Maternal	Cord	
Abdel Hameed et al., 2019 [34]	Cairo, Egypt	113	2016-2017	Serum	ICP-MS	0.70 ± 0.40	0.70 ± 0.30	Significant negative correlation between maternal Cd levels and 1-minute Apgar scores. Significantly higher Cd levels in maternal serum and cord serum in mothers passively exposed to tobacco smoke than in controls.
Al-Saleh et al., 2011 [12]	Al-Kharj area, Saudi Arabia	1578	2005-2006	Blood	ET-AAS	0.99 ± 0.31	0.78 ± 0.62	Only few newborns had Cd blood levels above the OSHA limit of 5 μg/L. Compared to the German reference value for non-smokers, 48.6% of mothers had a blood Cd level >1.0 μg/L.
Walker et al., 2006 [105]	Arctic, Canada	523	1994-1999	Blood	GF-AAS	0.43 ± 4.09	0.10 ± 0.61	Significantly higher Cd level in maternal blood than in cord blood. About 53% of all participants were smokers; Cd levels in maternal blood in moderate smokers (1-8 cigarettes per day) and heavy smokers (> 8

								cigarettes per day) were about 7.5-fold higher and 12.5-fold higher than non-smokers, respectively.
Koppen et al., 2009 [71]	Flanders, Belgium	1112	2002-2004	Blood	HR-ICP-MS	-	0.21 (0.19-0.23)	Maternal age and smoking habits did not affect cord blood Cd levels.<br>Significantly higher maternal blood Cd levels in women who gave birth in the summer than in women who gave birth in the winter.
Zhang et al., 2004 [75]	Hubei province, China	47	2002-2003	Blood	ICP-MS	0.80-25.2	0.02-1.48	Significantly lower Cd levels in cord blood than in maternal blood. Lower maternal blood copper (Cu) levels were significantly correlated with higher cord blood Cd levels.

Mokhtar et al., 2002 [158]	Cairo, Egypt	100	NP	Serum	GF-AAS	0.70 ± 0.30 (0.40-2.20)	0.70 ± 0.20 (0.20-1.50)	Significantly higher Cd levels in maternal serum (0.83 ± 0.31 μg/L) and cord serum (0.70 ± 0.19 μg/L) of women passively exposed to tobacco smoke than in controls (0.60 ± 0.14 and 0.50 ± 0.14 μg/L, respectively).<br>Negative correlation between 5-minute Apgar score and cord blood Cd level.

Adelouahab et al., 2010 [92]	Nancy and Poitiers, France	163	2002	Blood	AAS	0.98 ± 0.43	0.70 ± 0.40	No association between Cd levels in maternal blood or cord blood and MAO.
Vigeh et al., 2006 [167]	Tehran, Iran	365	2003-2004	Blood	ICP-MS	0.50 ± 0.30 (ND - 6.30)	0.35 ± 0.44 (ND-6.30)	Cord blood Cd levels were not significantly higher in PE cases than in the control group.
Eik Anda et al., 2007 [139]	Chuchki district, Russia	48	2001-2002	Whole blood and plasma	ET-AAS	1.21 ± 0.98	0.43 ± 0.72	Cadmium levels were 2.8-fold higher in maternal blood than in cord blood.
Rudge et al., 2009 [36]	South Africa	62	NP	Blood	ICP-MS	0.15 (0.04-0.89)	0.02 (ND-0.32)	No statistically significant correlation between Cd levels in maternal and cord blood.
Osman et al., 2000 [64]	Stockholm, Sweden	101	1994-1996	Blood	ICP-MS	0.16 (0.001-2.03)	0.02 (ND-0.08)	Statistically lower Cd level in core blood than in maternal blood (core blood contained 10% of the Cd measured in maternal blood). Significantly higher blood Cd level in pregnant women who smoked than in controls. Median birth weight of infants born to smoking mothers was approximately 200 g lower than that of nonsmoking mothers.

Ataniyazova et al., 2001 [68]	Karakalpakstan, Uzbekistan	45	NP	Blood	GF-AAS	0.15 (0.05-3.50)	0.12 (0.12-0.27)	No significant difference between maternal blood Cd level in 17 pregnant women and 28 controls.
Bocca et al., 2019 [13]	Tarragona, Spain	53	2016-2017	Blood	SF-ICP-MS	0.40 (0.30-2.50)	0.50 (0.20-0.90)	No significant difference between Cd levels in maternal blood and cord blood. No significant correlation between Cd levels in paired maternal and cord blood.
Arbuckle et al., 2016 [67]	10 cities across Canada	2001	2008-2011	Blood	ICP-MS	0.202	-	No significant difference between maternal blood Cd levels in the first and third trimesters of pregnancy.
Baeyens et al., 2014 [32]	Flanders	255	2007-2011	Blood	HR-ICP-MS	0.31	0.07	No significant correlation between Cd in cord blood and maternal age. Cadmium levels in cord blood samples were very low, indicating limited transport of Cd across the placenta. Significantly higher maternal Cd levels in mothers who smoked during pregnancy than in controls. This was not the case for cord blood Cd levels. Significant increase in maternal

								blood Cd levels with increasing smoking frequency category (never smoked, 0.273 µg/L;

								former smoker, 0.274 μg/L; less than daily, 0.286 μg/L; daily, 0.538 μg/L).
Jin et al., 2014 [47]	China	215	2005-2009	Blood	ICP-MS	0.47	-	Maternal blood Cd levels were higher in Yushe County (median: 0.54 μg/L) than in Daixian County (0.39 μg/L), which had a higher prevalence of NTDs.
Sun et al., 2014 [74]	Jiangsu province, eastern China	209	NP	Blood	ICP-MS	0.48	0.15	Significantly higher Cd levels in maternal blood than in cord blood. Significant correlation between Cd levels in maternal and cord blood. They stated that maternal Cd exposure could negatively affect the birth weight of the newborn.
Kopp et al., 2012 [65]	Bochum, Germany	50	2006	Blood	GF-AAS	0.34	-	Significantly higher maternal blood Cd levels in smokers (0.73 μg/L) than in non-smokers (0.29 μg/L). Effect was dose-dependent in terms of increasing Cd levels with increasing number of cigarettes smoked per day. No direct relation between increasing fetal Cd levels and

								increasing maternal Cd exposure.

Kim et al., 2015 [57]	South Korea	104	2013	Blood	ICP-MS	0.61 ± 1.51	0.01 ± 5.31	The geometric mean of blood Cd levels was significantly lower at birth than at one year of age.
Hansen et al., 2011 [40]	North Norway	211	2007-2009	Blood	ICP-MS	0.15	-	Cd levels in maternal blood of nonsmokers increased during pregnancy and from birth to 6 weeks postpartum.
Al-Saleh et al., 2014 [96]	Al-Kharj area, Saudi Arabia	1579	2005-2006	Blood	GF-AAS	0.98	0.70	Logistic regression models showed that 5-minute Apgar score, birth weight, and SGA were associated with cord blood Cd. Significant decrease in placental thickness with higher levels of Cd in maternal blood. As Cd levels in the placenta increased, placental thickness decreased significantly and umbilical cord length increased significantly.

García-Esquinas et al., 2013 [61]	Spain	140	2003-2004	Blood	GF-AAS	0.53	0.27	About 29% of cord blood samples exceeded the reference level of 0.5 μg/L for Cd established by the Human Biomonitoring Commission of the German Federal Agency. Cadmium levels were 22% higher in cord blood from mothers who smoked during pregnancy. Cord

								blood Cd levels above 0.29 μg/L were associated with lower 1-minute and 5-minute Apgar scores.
Cabrera-Rodríguez et al., 2018 [55]	Canary Islands, Spain	471	2015-2016	Blood	ICP-MS	-	0.01 ± 0.02	No significant differences in cord blood Cd levels in relation to smoking, likely due to the small number of smoking mothers included in the study.
de Assis Araujoet al., 2020 [39]	Rio de Janeiro, Brazil	140	2017-2018	Blood	ICP-MS	0.30	0.41	Significant positive correlation between Cd levels in maternal and cord blood.
Dursun et al., 2016 [141]	Ankara, Turkey	123	2006-2007	Blood	ICP-MS	-	Cd was detected in 24 samples (median value < LOD)	Cadmium in cord blood was significantly more common in mothers who consumed fish twice a week during pregnancy than in those who consumed it less than twice a week.

Fagerstedt et al., 2015 [43]	Stockholm, Swedish	330	2004-2007	Erythrocytes	ICP-MS	0.37	0.03	Cadmium levels in maternal erythrocytes were significantly higher in the anthroposophic lifestyle group than in the non-anthroposophic group. Since smokers were excluded from their study, the higher Cd levels

								were likely a consequence of a diet rich in plant foods and/or maternal Fe status.
Guy et al., 2018 [66]	Sô-Ava district, Benin	60	2015-2016	Blood	ICP-MS	0.35	0.20	Significantly higher Cd levels at delivery than during the first trimester of pregnancy. Significantly lower Cd levels in cord blood than in maternal blood.
Hu et al., 2015 [45]	Four cities (Beijing, Lanzhou, Taiyuan, and Xiamen), China	81	2011	Blood	ICP-MS	0.90	0.60	Significant difference between Cd levels in maternal blood and cord blood. No significant association between maternal or cord Cd level and birth weight.
Huang et al., 2021 [23]	Sirajikhan and Pabna Sadar Upazila, Bangladesh	745	2008-2011	Serum	ICP-MS	-	0.027 (0.027-0.151)	No significant association between cord serum Cd level and preterm birth.
Iijima et al., 2007 [69]	Tokyo, Japan	24	2005	Blood	ICP-MS	-	0.20	Significant negative correlation between cord blood Cd level and cord blood TSH level, indicating that *in utero* Cd exposure affected the thyroid hormone status of

								newborns.

Iwai-Shimada et al., 2019 [46]	Tohoku region, Japan	687	2001-2006	Blood	ICP-MS	1.18 (0.74-1.79)	0.53 (0.10-1.25)	Significantly lower Cd level in cord blood than in maternal blood.
Kippler et al., 2010 [70]	Matlab, Bangladesh	44	2004-2007	Erythrocytes	ICP-MS	-	0.16	Metallothionein (MT) expression was significantly higher in placentas with high Cd levels compared to in those with low Cd levels. Positive relationship between MT expression and the placenta:cord blood Cd ratio. No association of Cd level in cord blood and fetal growth.
Kot et al., 2019 [72]	Szczecin, Poland	83	NP	Blood	ICP-OES		0.008 (0.004-0.025)	No statistically significant differences between Cd levels in the placenta, fetal membranes, and/or cord blood.
Kucukaydin et al., 2018 [62]	Turkey	68	2008-2009	Serum	FAES	46.0 ± 90.0	22.0 ± 22.0	No significant difference between Cd levels in maternal blood and cord blood. Cadmium levels in both matrices did not differ in women with PPROM (n = 35) compared to women without PPROM (n = 33).

Lee et al., 2021 [153]	Sirajdikhan and Pabna Sadar Upazilas, Bangladesh	1088	2008-2011	Blood	ICP-MS	-	0.60 ± 1.20	Statistical association of increasing Cd levels in cord blood with lower mean birth weight and with mean

								head circumference after correction for covariates.
Parajuli et al., 2012 [50]	Terai region, Nepal	100	2008	Blood	ICP-MS	-	0.23 (0.23 - 2.63)	Positive correlation between cord blood Cd level and maternal age. No correlation between cord blood Cd level and tobacco smoking.
Rahbar et al., 2015 [44]	Kingston, Jamaica	100	2011	Blood	ICP-MS	-	0.07	No significant association between cord blood Cd level and head circumference.
Rollin et al., 2009 [30]	Seven geographical areas, South Africa	96	2005-2007	Blood	ICP-MS	0.10 (0.05- 0.25)	0.04 (0.01- 0.10)	No correlation between Cd levels in maternal and cord blood. The highest (and statistically significant compared to all other locations studied) Cd levels were in maternal blood at the Atlantic location. Significant differences in maternal blood Cd levels between rural and industrial locations. Tobacco smoking was a confounding factor for high Cd levels.
Sakamoto et al.,	Munakata City,	81	NP	Erythrocytes	ICP-MS	1.97 ± 0.72	0.22 ± 0.20	Significantly lower Cd levels in cord erythrocytes than in maternal erythrocytes. Significant

2010<br>[115]	Fukuoka,<br>Japan							positive

								correlation between Cd levels in maternal and cord erythrocytes.
Sekovanić et al., 2018 [164]	Zagreb and Zadar, Croatia	268	2008-2010	Blood	ICP-MS	0.42 ± 0.15	0.05 ± 0.03	Tobacco smoking was a significant predictor of increased levels of Cd and MT2 in maternal blood. Significantly higher Cd levels in both maternal and cord blood of smokers than in controls.
Stojsavljević et al., 2022 [166]	Serbia	125	2020-2021	Plasma	ICP-MS	0.47 ± 0.31	0.31 ± 0.15	No significant difference between Cd levels in maternal blood and in cord blood.
Tang et al., 2016 [81]	Shengsi Island, China	103	2011-2012	Serum	ICP-MS	-	10.69 ± 14.63	No significant associations between Cd exposure and birth weight, height, head circumference, or gestational age in after adjusting for numerous covariates.
Zheng et al., 2014 [52]	Xiamen, China	1106	2010	Serum	ICP-MS	-	0.29 (0.15-0.51)	No significant differences in serum Cd levels between the group with adverse pregnancy outcomes and controls.
Zhou et al., 2020 [41]	Beijing, China	52	2013-2014	Blood clots	ICP-MS/MS	10.10 ± 11.98	0.36 ± 0.13	Significantly lower Cd levels in cord blood clots than in maternal blood clots. The placenta could

								act<br>effectively as a barrier against Cd and

								protect the fetus from its detrimental effects, while it had almost no role in preventing the transport of As and Pb.
Dahiri et al., 2023 [42]	Seville, Spain	100	2020-2021	Blood	ICP-MS	0.82	0.73	No significant difference between Cd levels in maternal and in cord blood.
Gu et al., 2022 [31]	Peking, China	48	NP	Plasma	ICP-MS	0.97 (0.65-1.29)	0.93 (0.71-1.17)	No significant difference between Cd levels in maternal and in cord blood.
Luo et al., 2024 [129]	Zhejiang province, China	48	2018-2019	Blood	ICP-MS	0.60 (0.40-1.10)	0.10 (< 0.10-0.10)	Positive correlation between Cd levels in maternal and in cord blood.
Park et al., 2022 [73]	Ulsan, Ehwa, and Dankook, South Korea	384	2006-2010	Blood	GF-AAS	1.50 (0.47-3.37)	0.64 (0.03–1.62)	Prenatal Cd exposure disrupts several DNA methylation sites in neonatal cord blood in a time-specific manner, indicating that the fetus could be susceptible to Cd-induced epigenetic modifications based on the trimester of pregnancy.

Wang et al., 2022 [48]	Eight provinces of China	303 CHD cases and 303 healthy	2016-2020	Plasma	ICP-MS	0.12 (0.03-0.26)	-	Significantly higher Cd levels in cases with CHD than in controls. No significant association of Cd levels with increased risk of CHDs.

		controls						
Zinia et al., 2023 [82]	Korea	5,215	2015-2019	Blood	ICP-MS	0.70 ± 0.32	0.24 ± 0.12	Significantly higher maternal blood Cd levels in late pregnancy than in early pregnancy. Significant associations between maternal blood Cd levels in both early and late pregnancy with birth weight.<br>Statistical association between maternal blood Cd levels in early pregnancy and large birth weight for gestational age.

Abbreviations: *N* – total number of participants; NP – not presented; ND – not detected; LOD – limit of detection; ICP-MS – inductively coupled plasma mass spectrometry; SF – sector field; HR – high resolution; MS/MS – tandem mass spectrometry; AAS – atomic absorption spectrophotometry; ET- electrothermal; GF – graphite furnace; ICP-OES- inductively coupled plasma optical emission spectrometry; FAES – flame atomic emission spectroscopy; OSHA - Occupational Safety and Health Administration; MAO – monoamine oxidase TSH – thyroid stimulating hormone; PPROM – preterm premature rupture of membranes; NDTs – neural tube defects; SGA – small for gestational age; AGA – appropriate for gestational age; LGA – large for gestational age; PE – preeclampsia; GDM – gestational diabetes mellitus; IUGR – intrauterine growth restriction; CHDs – congenital heart defects.

Table S3. Lead (Pb) levels in maternal and cord blood of non-occupationally exposed healthy pregnant women worldwide (μg/L).

Reference	Country	*N*	Blood collection time period	Biological material	Analytical technique	Pb levels (μg/L)		Main findings
						Maternal	Cord	
Al-Saleh et al., 2011 [12]	Al-Kharj area, Saudi Arabia	1578	2005-2006	Blood	ET-AAS	25.5 ± 16.4	21.4 ± 16.9	A very small number of maternal blood and cord blood samples had Pb levels above the CDC limit of 100 μg/L. Maternal blood Pb level was 2.3% higher than the German reference value for women of 70 μg/L.
Amaral et al., 2010 [138]	Sao Paulo, Brazil	120	NP	Blood and serum	ICP-MS	17.4 ± 0.90	11.9 ± 0.62	Significantly higher Pb levels in maternal blood than in cord blood and in maternal serum than in cord blood.<br>Positive correlations between Pb levels in maternal and cord blood, and between Pb levels in maternal and cord serum; both comparisons in matched pregnant women.
Koppen et al., 2009 [71]	Flanders, Belgium	1112	2002-2004	Blood	HR-ICP-MS	-	14.7 (14-155)	No effect of maternal age or smoking habits on cord blood Pb levels.<br>Significantly higher blood Pb levels

								in<br>women who gave birth in summer

								than in women who gave birth in winter.
Walker et al., 2006 [105]	Arctic, Canada	523	1994-1999	Blood	GF-AAS	20.58 ± 17.89	15.2 ± 14.4	Significantly higher Pb levels in maternal blood than in cord blood. Significantly higher maternal blood Pb levels in both Dene/Métis (30.9 μg/L) and Inuit mothers (31.6 μg/L) than in Caucasian mothers (20.6 μg/L).
Levesque et al., 2003 [99]	Quebec, Canada	475	1993-1996	Blood	GF-AAS	-	39.3 (2.07-271)	Significant reduction in cord blood Pb levels following a public health intervention to reduce the use of Pb shot.
Kawata et al., 2006 [148]	Yunnan province, China	100	NP	Blood	GF-AAS	53.1 ± 27.9	67.3 ± 29.6	Significant positive correlation between Pb levels in maternal and cord blood. Maternal occupational exposure, maternal consumption of homemade dehydrated vegetables, and maternal residence in Kunming was significantly associated with higher cord blood Pb levels compared to controls. Significant association between high cord blood Pb levels and

								frequent use by the mother of cutlery that had colorful patterns inside.
Adelouahab et al., 2010 [92]	Nancy and Poitiers, France	163	2002	Blood	AAS	0.18 ± 0.12	0.15 ± 0.09	Inverse correlation between cord blood Pb levels and MAO.
Bjerregaard and Hansen, 2000 [121]	Disko Bay area, Greenland	180	1994-1996	Blood	GF-AAS	41.5 ± 34.5	32.6 ± 18.0	Significantly lower Pb levels in cord blood than in maternal blood.
Raghunath et al., 2000 [159]	Mumbai, India	148	1993-1997	Blood	DPASV	64.0 ± 16.9	51.0 ± 19.6	Prenatal Pb exposure in India was 2-3 times higher than in Canada or Italy.
Al-Jawadi et al., 2009 [137]	Mousel, Iraq	350	2006-2007	Blood	EC sensor	32.6 ± 19.1	22.9 ± 21.1	Significant difference in Pb levels between maternal and cord blood. Strong positive correlation between Pb levels in maternal and cord blood. Low pregnancy rates, smoking, and hemoglobin levels < 11 gm/dL were significant predictors of high maternal blood Pb levels.

Janjua et al., 2008 [146]	Karachi, Pakistan	540	2005	Blood	GF-AAS	-	108. ± 57.0	Women who consumed less than 58.5 mg of elemental Fe per day during pregnancy had cord blood Pb levels of about 100 μg/L compared to women who had higher Fe intake and lower cord blood Pb levels. Pregnant women

								who used surma (an eye cosmetic) daily had higher cord blood Pb levels than those who used it less frequently.
Jedrychowski et al., 2009 [147]	Krakow, Poland	444	2001-2004	Blood	ICP-MS	-	12.3 (4.40-69.0)	Borderline significant detrimental effect of prenatal Pb exposure on mental development index (MDI) scores at 12 months of age. Follow-up testing of children at 24 months of age showed a significant inverse association between mental function and Pb exposure. Significant cognitive deficits were also confirmed at 36 months of age. Girls' mental function scores were better than boys', and the effect of maternal education level remained highly significant on the mental function of the 3-year-olds.
Reis et al., 2007 [161]	Lisbon, Portugal	145	NP	Blood	AAS	71.0 ± 28.0	70.0 ± 29.0	No significant difference in Pb levels between maternal blood and cord blood. No statistical correlation between maternal blood Pb level and maternal age. Positive correlation between Pb

								levels in maternal and cord blood. This could be explained by the diffusion of Pb across the

								placenta, providing evidence of Pb exposure *in utero*.
Eik Anda et al., 2007 [139]	Chuchki district, Russia	48	2001-2002	Blood and plasma	ET-AAS	50.0 ± 30.0	45.0 ± 30.0	Lead levels were 1.1-fold higher in maternal blood than in cord blood.
Rudge et al., 2009 [36]	South Africa	62	NP	Blood	ICP-MS	23.0 (6.01-161)	15.4 (1.40-95.1)	Lead crosses the placental barrier by diffusion.
Osman et al., 2000 [64]	Stockholm, Sweden	101	1994-1996	Blood	ICP-MS	11.4 (2.07-47.6)	11.2 (0.89-122)	No significant difference between Pb levels in cord blood and in maternal blood. Statistically significant negative associations between cord blood Pb levels and the weight, height, and head circumference of newborns.
Lin et al., 2010 [155]	Taiwan	308	2004-2005	Blood	ICP-MS	15.8 ± 11.1	12.9 ± 7.20	Positive correlation between Pb levels in maternal blood and cord blood.<br>Cord blood Pb levels were significantly lower if the mother had higher blood Zn or Mn levels; therefore, maternal Zn and Mn appear to reduce the transfer of Pb across the placenta.

Kirel et al., 2005 [150]	Eskisehir, Turkey	143	2001-2002	Blood	GF-AAS	28.0 ± 15.0	16.5 ± 14.0	Strong correlation between Pb levels in maternal blood and cord blood. Correlation between maternal blood Pb level and newborn head circumference.
Schell et al., 2003 [163]	New York, USA	220	1986-1998	Blood	ET-AAS	22.0 ± 17.2	16.0 ± 17.8	Strong positive association between Pb levels in maternal blood and cord blood. More than 50% of mothers had dietary intakes below the recommended daily allowances of Zn, Ca, Fe, and vitamin D.<br><br>Negative association of cord blood Pb levels with maternal nutritional status, measured weight gain and arm circumference.<br><br>Negative association between cord blood Pb level and dietary Fe and vitamin D intakes.
Harville et al., 2005 [143]	Pittsburg, USA	159	1992-1995	Blood	ET-AAS	19.6 ± 8.40	16.5 ± 7.60	Association between higher blood pressure with relatively higher levels of Pb in cord blood. No association between maternal or cord blood Pb levels with

								smoking, physical exertion, or Ca intake.

Ataniyazova et al., 2001 [68]	Karakalpakstan, Uzbekistan	45	NP	Blood	GF-AAS	61.5 (20.0-499)	47.8 (23.1-836)	No significant difference between blood Pb levels in 17 pregnant women and 28 non-pregnant women.
Bocca et al., 2019 [13]	Tarragona, Spain	53	2016-2017	Blood	SF-ICP-MS	12.0 (5.20-41.0)	7.90 (2.80–32.0)	Lead levels in maternal blood were significantly higher than in cord blood, suggesting that the placenta could modulate Pb transfer. Significantly higher maternal blood Pb levels at birth than in the first trimester.
Arbuckle et al., 2016 [67]	10 cities across Canada	2001	2008-2011	Blood	ICP-MS	5.59	7.67	Significantly higher Pb levels in cord blood than in maternal blood. Significant changes in maternal blood Pb levels occurred during pregnancy. Speculated that increasing dietary Ca and vitamin D intake during pregnancy could reduce maternal blood Pb and reduce cord blood Pb levels.

Baeyens et al., 2014 [32]	Flanders	255	2007-2011	Blood	HR-ICP-MS	11.1	8.60	Strong positive correlation between Pb levels in maternal and cord blood, indicating Pb transport across the placenta. Significant positive correlation between cord blood Pb level and maternal age. This indicates

								that the placenta leaks Pb and that Pb accumulates with the mother's age. Significantly higher maternal blood Pb levels in those who smoked during pregnancy than in controls. A similar trend was observed in cord blood, but without statistical significance.
Sun et al., 2014 [74]	Jiangsu province, eastern China	209	NP	Blood	ICP-MS	40.5	32.3	Significantly higher levels of Pb in maternal blood than in cord blood. Significant correlation between Pb levels in maternal and cord blood.
Kopp et al., 2012 [65]	Bochum, Germany	50	2006	Blood	GF-AAS	11.5	10.3	No significant differences between Pb levels in maternal blood and in cord blood. Significant correlation between cord blood Pb level and maternal Pb exposure.
Kim et al. 2015 [57]	South Korea	104	2013	Blood	ICP-MS	10.2 ± 13.9	7.10 ± 14.2	No significant differences between Pb levels in maternal blood at the second trimester, delivery, and 1 year postpartum. Approximately 72%-76% of maternal Pb was transferred to newborns.

Hansen et al., 2011 [40]	North Norway	211	2007-2009	Blood	ICP-MS	7.50	-	Maternal blood Pb levels increased during pregnancy and from birth to 6 weeks postpartum.
Al-Saleh et al., 2014 [96]	Al-Kharj area, Saudi Arabia	1579	2005-2006	Blood	GF-AAS	25.4	20.5	Maternal blood Pb level affected the thickness of the placenta.
García-Esquinas et al., 2013 [61]	Spain	140	2003-2004	Blood	GF-AAS	19.8	14.1	Higher Pb levels (15%) in newborns from mothers who smoked during pregnancy than in controls.
Cabrera-Rodríguez et al., 2018 [55]	Canary Islands, Spain	471	2015-2016	Blood	ICP-MS		1.62 ± 2.26	No significant differences between cord blood Pb levels in relation to smoking, likely due to the small number of smoking mothers.
de Assis Araujo et al., 2020 [39]	Rio de Janeiro, Brazil	140	2017-2018	Blood	ICP-MS	37.4	38.5	Significant positive correlation between Pb levels in maternal and cord blood.
Dursun et al., 2016 [141]	Ankara, Turkey	123	2006-2007	Blood	ICP-MS		16.6 ± 16.0	Cord blood Pb levels were higher in mothers older than 35 years than in mothers younger than 35 years (22.0 ± 23.4 versus 15.2 ± 13.1 μg/L).

Fagerstedt et al., 2015 [43]	Stockholm, Swedish	330	2004-2007	Erythrocytes	ICP-MS	15.8	7.85	Lead levels were about 30% higher in maternal and cord erythrocytes of mothers with an anthroposophic

								lifestyle than in mothers with a non-anthroposophic lifestyle.
Guy et al., 2018 [66]	Sô-Ava district, Benin	60	2015-2016	Blood	ICP-MS	38.0	26.4	Significantly lower Pb levels in cord blood than in maternal blood. Significantly higher Pb levels at delivery than during the first trimester of pregnancy. During the first trimester, 8.3% of women had blood Pb levels > 50 µg/L.
Hu et al., 2015 [45]	Four cities (Beijing, Lanzhou, Taiyuan, and Xiamen), China	81	2011	Blood	ICP-MS	23.1	22.0	No significant difference between Pb levels in maternal blood and in cord blood. No significant associations between maternal blood and cord blood Pb level and birth weight.
Huang et al., 2021 [23]	Sirajikhan and Pabna Sadar Upazila, Bangladesh	745	2008-2011	Serum	ICP-MS		4.075 (2.585–7.631)	No significant association between Pb level in cord serum and preterm birth.
Irwinda et al., 2019 [98]	Jakarta, Indonesia	51	2017	Serum	ICP-MS	32.5 (15.0 - 76.0)	23.5 (08.00 - 57.0)	No significant association between Pb levels in maternal serum or in cord serum with preterm birth.

Iwai-Shimada et al., 2019 [46]	Tohoku region, Japan	687	2001-2006	Blood	ICP-MS	10.83 (8.65-13.50)	9.89 (8.02-12.48)	Significantly lower Pb levels in cord blood than in maternal blood.
Kayaalti et al., 2015 [149]	Ankara, Turkey	93	2011	Blood	GF-AAS			Mothers with the H63D gene variant (HFE gene; human hemochromatosis protein) have higher levels of Pb in the cord blood of their newborns than in mothers without H63D. Significantly higher Pb levels in maternal blood and cord blood in mothers with HD+DD genotypes than in mothers with the HH genotype.
Kot et al., 2021 [151]	Gryfino and Kutno, Poland	136	2014-2015	Blood	ICP-OES	20.0 ± 10.0	20.0 ± 10.0	No significant difference between Pb levels in maternal blood and in cord blood. In their previous study (Kot et al., 2019), the authors found significantly higher levels of Pb in the umbilical cord tissue than in the placenta and fetal membrane.

Kucukaydin et al., 2018 [62]	Turkey	68	2008-2009	Serum	FAES	154.1 ± 98.5	151.9 ± 122.8	No significant difference between Pb level in maternal blood and in cord blood. No significant difference between Pb levels in maternal and cord blood matrices from women with

								PPROM (n = 35) compared to women without PPROM (n = 33).
Ladele et al., 2019 [84]	Lagos, Nigeria	400	NP	Blood	AAS	64.3	39.2	Strong positive correlation between Pb levels in maternal blood and cord blood. Significant correlation between Ca supplements use during pregnancy and lower maternal blood Pb levels. Correlation of high cord blood Pb levels with recent painting and renovation of residential properties.
Lee et al., 2021 [153]	Sirajdikhan and Pabna Sadar Upazilas, Bangladesh	1088	2008-2011	Blood	ICP-MS	-	46.3 ± 53.8	No significant relation between cord blood Pb level and birth weight.

Li et al., 2021 [154]	Shenyang, China	121	2019-2020	Blood	GF-AAS	24.66	22.90	Cord blood Pb levels in Chinese residents are still higher than in most developed countries. Significant correlations between cord blood Pb levels and the third trimester of pregnancy. Lower risk ratio for cord blood Pb levels ≥ 20 μg/L in pregnant women who were not exposed to secondhand smoke than in exposed pregnant women

McKeating et al., 2020 [157]	Queensland, Australia	2879	2006-2010	Blood	ICP-MS		4.68 ± 2.23	Significantly lower cord blood Pb levels in preterm pregnancies (2.79 μg/L) than in controls (4.68 μg/L), PE (5.32 μg/L), and GDM (8.27 μg/L).
Parajuli et al., 2012 [50]	Terai region, Nepal	100	2008	Blood	ICP-MS	-	20.6 (6.83-221)	No association between cord blood Pb level and socioeconomic status, maternal age, living environment, or smoking status.
Rahbar et al., 2015 [44]	Kingston, Jamaica	100	2011	Blood	ICP-MS	-	6.00	Significant association between cord blood Pb level and head circumference.
Reddy et al., 2014 [160]	Hyderabad, India	60	NP	Blood	GF-AAS	13.5 ± 6.97	8.5 ± 2.85	Significantly higher maternal blood Pb levels in urban women after childbirth than in rural counterparts. Blood Pb levels in women of reproductive age have been declining in recent decades, but low blood Pb levels are still a cause for caution.
Rollin et al., 2009 [30]	Seven geographical areas, South Africa	96	2005-2007	Blood	ICP-MS	20.9 (7.4-50.3)	24.0 (15-87)	Significant correlation between Pb levels in maternal and cord blood. Significantly higher Pb levels in urban area than in other locations.

Sakamoto et al., 2010 [115]	Munakata City, Fukuoka, Japan	81	NP	Erythrocytes	ICP-MS	26.4 ± 9.74	13.2 ± 4.12	Significantly lower Pb levels in cord erythrocytes than in maternal erythrocytes. Significant positive correlation between Pb levels in maternal and cord erythrocytes.
Sekovanić et al., 2018 [164]	Zagreb and Zadar, Croatia	268	2008-2010	Blood	ICP-MS	13.0 ± 6.90	7.70 ± 5.50	Significantly higher Pb levels in maternal and cord blood of smokers than in non-smokers.
Stojsavljević et al., 2022 [163]	Serbia	125	2020-2021	Plasma	ICP-MS	4.92 ± 4.01	0.94 ± 0.80	Significantly higher Pb levels in maternal plasma than in cord plasma.
Tang et al., 2016 [81]	Shengsi Island, China	103	2011-2012	Serum	ICP-MS	-	128 ± 286	Significant associations between a one-unit increase in serum Pb level (μg/L) and a 0.29 cm decrease in birth height and a 0.22 cm decrease in head circumference.
Zheng et al. 2014 [52]	Xiamen, China	1106	2010	Serum	ICP-MS	-	25.1 (17.6-50.6)	No significant differences between Pb levels in the group with adverse pregnancy outcomes and the control group.
Zhou et al., 2019 [41]	Beijing, China	52	2013-2014	Blood clots	ICP-MS/MS	130 ± 122	94.7 ± 58.6	Significantly lower Pb levels in cord blood clots than in maternal blood clots. Significant negative correlation

								between Pb levels in maternal and cord blood clots.
Dahiri et al. 2023 [42]	Seville, Spain	100	2020-2021	Blood	ICP-MS	10.2	9.51	No significant difference between Pb levels in maternal and cord blood.
Grzesik-Gąsior et al., 2023 [142]	Lublin Province, Poland	134	2020-2021	Blood	ICP-OES	-	26.25 ± 9.32	No significant correlations between cord blood Pb levels and anthropometric parameters (birth weight, birth length, head circumference, and chest circumference).
Gu et al., 2022 [31]	Peking, China	48	NP	Plasma	ICP-MS	0.87 (0.63-1.37)	0.78 (0.63-1.05)	No significant difference between Pb levels in maternal and cord blood. Lead levels decreased significantly from the first to the third trimester of pregnancy.
Huang et al., 2023 [97]	Lanzhou, China	194	2010-2012	Blood	ICP-MS	64.5	39.1	Associations between high Pb levels both in maternal blood and in cord blood with an increased risk of CHDs.
Luo et al., 2024 [49]	Zhejiang province, China	48	2018-2019	Blood	ICP-MS	12.5 (9.2-14.8)	8.80 (6.00-13.2)	Positive correlation between Pb levels in cord and maternal blood. Positive association between maternal blood Pb levels and passive smoking.

Mahdi et al., 2023 [156]	Lucknow, India	200	NP	Blood	ICP-OES	10.4 ± 6.36	10.7 ± 8.27	About 38.5% of cord blood samples had Pb levels above 50 µg/L. Strong positive correlation between Pb levels in maternal and cord blood. Significant relation of high maternal blood Pb levels with recent house painting and living near traffic congestion. Iron and Ca deficiencies observed in women with high Pb levels (> 50 µg/L).
Vigeh et al., 2006 [167]	Tehran, Iran	365	2003-2004	Blood	ICP-MS	4.82 ± 2.22	3.52 ± 2.09	Significantly higher cord blood Pb levels in PE cases than in controls. A one-unit (µg/L) increase in cord blood Pb level leads to a 13.0-fold increase in the risk of PE.
Wang et al., 2022 [48]	Eight provinces of China	303 CHD cases and 303 healthy controls	2016-2020	Plasma	ICP-MS	2.16 (1.13-3.92)	-	No significant differences between Pb levels in maternal plasma from CHD cases and controls. Marginal positive association of maternal plasma Pb levels with increased risk of CHD.

Zhang et al., 2023 [168]	Shanghai, China	100	2013-2016	Blood	ICP-MS	12.6 ± 4.98	14.5 ± 5.86	Lead levels in maternal blood were notably lower during early, mid, and late pregnancy than before conception.

								Positive correlation between Pb levels in late pregnancy maternal blood and cord blood in the newborn.

Abbreviations: *N* – total number of participants; NP – not presented; ND – not detected; LOD – limit of detection; CDC – Centers for Disease Control and Prevention; Zn – zinc, Mn – manganese; Fe – iron; Ca – calcium; ICP-MS – inductively coupled plasma mass spectrometry; SF – sector field; HR – high resolution; MS/MS – tandem mass spectrometry; AAS – atomic absorption spectrophotometry; ET – electrothermal; DPASV – Differential pulse anodic stripping voltammetry; ICP-OES – inductively coupled plasma optical emission spectrometry; FAES – flame atomic emission spectroscopy; GF – graphite furnace; MAO – monoamine oxidase; TSH- thyroid stimulating hormone; PPROM – preterm premature rupture of membranes; NDTs – neural tube defects; SGA – small for gestational age; AGA – appropriate for gestational age; LGA – large for gestational age; PE – preeclampsia; GDM – gestational diabetes mellitus; IUGR – intrauterine growth restriction; CHDs – congenital heart defects.

Table S4. Mercury (Hg) levels in maternal and cord blood of non-occupationally exposed healthy pregnant women worldwide (µg/L).

Reference	Country	*N*	Blood collection time period	Biological material	Analytical technique	Hg levels (µg/L)		Main findings
						Maternal	Cord	
Abdel Hameed et al., 2019 [34]	Cairo, Egypt	113	2016-2017	Serum	ICP-MS	28.8 ± 11.8	15.65 ± 5.90	Significant positive correlation between Hg levels in maternal and cord blood.<br>No significant differences in Hg levels in maternal blood or cord blood depending on diet.
Al-Saleh et al., 2011 [12]	Al-Kharj area, Saudi Arabia	1578	2005-2006	Blood	HG-AAS	1.92 ± 2.92	2.57 ± 2.51	About 11% of maternal blood and 13% of cord blood had Hg levels above >5.8 µg/L EPA reference dose. Almost 49% of women had Hg levels >2.0 µg/L, which is the German reference value for those who consume fish ≤ 3 times per month.
Walker et al., 2006 [105]	Arctic, Canada	523	1994-1999	Blood (Caucasian group)	CV-AAS	iHg: 0.39 ± 0.66<br>MeHg: 0.69 ± 1.97	iHg: 0.40 ± 0.55<br>MeHg: 1.14 ± 1.33	Significantly higher tHg and MeHg levels in cord blood than in maternal blood. No significant difference between iHg levels in maternal and cord blood. They suggest that there are highly significant correlations between paired maternal blood and cord blood levels for tHg and MeHg, indicating

								that either maternal blood or cord blood

						tHg: 0.87 ± 1.95	tHg: 1.22 ± 2.80	can be used as biomarkers of prenatal Hg exposure, especially during the third trimester. Note: MeHg was calculated as the difference between tHg and iHg.
Santos et al., 2007 [132]	Itaituba, Pará State, Brazil	1510	2000-2002	Erythrocytes	CV-AAS	11.5	16.7	Divided mothers into 7 groups (from 11 to 47 years old). Highest Hg levels were in the 31 to 40 age group in mothers and their newborns, at 14.4 μg/L and 21.9 μg/L, respectively. In all age groups, significantly higher Hg levels in cord erythrocytes than in maternal erythrocytes (all age groups).<br><br>Strong positive correlation between Hg levels in maternal and cord erythrocytes.
Morrissette et al., 2004 [129]	Southwest Quebec, Canada	159	NP	Blood	CV-AAS	0.61 (ND - 1.20)	0.69 (ND - 1.60)	Significant drop in maternal Hg levels between the second and third trimesters. Significantly higher Hg levels in cord blood than in maternal blood. Fish consumption before and during pregnancy explained 26% and 20% of the variance in cord blood Hg, respectively.

Gao et al., 2007 [128]	Zhoushan city, China	417	2004	Blood	CV-AAS	-	5.58 (3.96-7.82)	About 70% participants had cord blood Hg levels above the US-EPA recommended blood level of 5.80 μg/L. Frequency of fish consumption was related to cord blood Hg

								levels. Greater prenatal Hg exposure was related to lower behavioral abilities in males, but not in females.
Dewailly et al., 2008 [126]	French Polynesia	241	2005-2006	Blood	ICP-MS	-	13.0 (0.05-48.1)	About 83% participants had cord blood Hg levels above the US-EPA recommended blood level of 5.80 μg/L. Among fish species, tuna contributed the most to Hg exposure. High levels of Se and LC-PUFA could mitigate the potential risk of prenatal Hg exposure. Due to high fish consumption by mothers, Polynesian babies are prenatally exposed to high doses of Hg.
Bjerregaard and Hansen, 2000 [121]	Disko Bay area, Greenland	180	1994-1996	Blood	AAS	16.8 ± 13.6	35.6 ± 32.1	Significantly higher Hg levels in cord blood than in maternal blood. Significant increases in Hg levels in blood with maternal age and with consumption of marine mammals.

Fok et al., 2007 [127]	Hong Kong, China	1057	2000-2001	Blood	FIMS-400 Flow Injection Mercury System	4.94 (3.65-6.88)	8.83 (6.34-12.4)	Significant difference between Hg levels in maternal blood and cord blood. Strong positive correlation between Hg levels of these two blood compartments. About 21.5% participants had Hg levels less than 5.80 µg/L. Higher maternal fish consumption and maternal age related to higher cord blood Hg levels. Consumption

								of marine fish increased cord blood Hg levels more than freshwater fish (5.09%/kg vs. 2.86%/kg). Female babies, maternal alcohol consumption, and greater maternal height associated with lower cord blood Hg levels.
Sakamoto et al., 2007 [109]	Three districts, Japan	116	NP	Blood	CVAAS (for tHg) GC-ECD (for MeHg)	tHg: 5.18 (3.63-7.34) MeHg: 4.77 (3.5-6.54)	tHg: 9.81 (6.96-13.6) MeHg: 9.32 (6.56-13.4)	More than 90% of the Hg in cord tissue, cord blood, and maternal blood was MeHg. Total Hg and MeHg in cord blood were about twice as high as in maternal blood. Strong correlation between both tHg and MeHg in cord tissue and cord blood Hg level.
Palkovicova et al., 2008 [130]	Slovakia	99	NP	Blood	Mercury analyzer AMA-254	0.63 (0.14-2.90)	0.80 (0.15-2.54)	Strong positive correlation between Hg levels in maternal and cord blood. Significant relationships between cord blood Hg levels and the number of maternal amalgam dental fillings and with the number of years since the last filling

Ramon et al., 2008 [131]	Valencia, Spain	249	2005-2006	Blood	AMA-254	-	9.90 (9.00-10.8)	About 75% of cord blood had Hg levels above 5.80 μg/L. Women who consumed a serving of large fatty fish, lean fish, or mixed fried fish two or more times per

					Mercury analyzed			week had average cord blood Hg levels 1.6, 1.4, and 1.3 times higher, respectively, than those who rarely or never consumed fish.
Rudge et al., 2009 [36]	South Africa	62	NP	Blood	ICP-MS	0.65 (0.10-8.80)	1.20 (0.10-9.70)	Mercury levels in cord blood were almost twice as high as that in maternal blood, indicating that the fetus may act as a “filter” for maternal Hg levels during pregnancy.
Ask et al., 2002 [140]	Stockholm, Sweden	119	NP	Blood	CV-AFS	oHg: 0.73 (0.19-2.10); iHg: 0.32 (0.03-1.20)	oHg: 1.40 (0.26-3.80); iHg: 0.34 (0.09-0.79)	Significant associations between both MeHg and Se levels in both maternal and cord blood, and these were more pronounced in freshwater fish consumers.
Hsu et al., 2007 [144]	Taipei, Twain	65	2004-2005	Blood	HG-310 mercury analyzer	9.10 ± 0.40	10.0 ± 0.55	Significantly higher Hg levels in cord blood than in maternal blood. Positive correlation between Hg levels in the two blood compartments. About 90% of the mothers tested had Hg levels > 5.80

								μg/L.<br>Significant association of fish consumption

								during pregnancy with maternal and cord blood Hg levels.
Unuvar et al., 2007 [122]	Istanbul, Turkey	143	2004-2006	Blood	CV-AAS	0.38 ± 0.50	0.50 ± 0.64	Maternal blood Hg levels generally lower than 5 μg/L. Significant correlation between Hg levels in maternal and cord blood. Main risk factors affecting Hg levels were consuming fish meal more than twice a week and having amalgam dental fillings.<br>Since mothers regularly ate vegetables every day, the low Hg levels could be linked to this.
Lederman et al., 2008 [152]	New York, USA	163	2001-2002	Blood	CV-AAS	2.29 ± 2.33	5.05 ± 6.64	Cord blood Hg levels were more than twice as high as maternal levels. Strong correlation between Hg levels in maternal and cord blood. Significantly higher maternal and cord blood Hg levels in women who consumed fish/seafood during pregnancy than in controls. No significant association between maternal or cord blood Hg levels with birth outcomes. Association of high Hg levels in cord blood with reduced psychomotor development

								(PDI) at 36 months and reduced performance, verbal skills, and full IQ at 48 months.

Sato et al., 2006 [162]	Hawaii, USA	275	2004-2005	Blood	NP	-	4.82 ± 3.40	About 28.5% participants had Hg levels above 5.80 μg/L. Significant association between the amounts of fish consumed during pregnancy and level of Hg in cord blood.
Bocca et al., 2019 [13]	Tarragona, Spain	53	2016-2017	Blood	SF-ICP-MS	1.80 (0.50-9.00)	2.80 (0.70-8.70)	Mercury in cord blood (2.80 μg/L) was approximately twice as high as in maternal blood (1.80 μg/L), which could indicate transplacental transfer of Hg from mother to fetus.
Arbuckle et al., 2016 [67]	10 cities across Canada	2001	2008-2011	Blood	ICP-MS	0.56	0.80	Significantly higher Hg levels in cord blood than in maternal blood. Significant differences in maternal blood Hg levels during pregnancy.
Jin et al., 2014 [47]	China	215	2005-2009	Blood	ICP-MS	0.26	-	Women who consumed fish three or more times a week had blood Hg levels that were about 35% higher than women who consumed fish one to two times a week, who in turn showed Hg levels that were 29% higher than women who consumed fish once a week.
Kopp et al., 2012 [65]	Bochum, Germany	50	2006	Blood	CV-AFS	0.44	1.48	Mercury levels in cord blood were about three times higher than Hg levels in the

								mothers. Increasing fetal Hg levels directly related to increasing maternal Hg exposure.
Kim et al., 2015 [57]	South Korea	104	2013	Blood	AAS (for tHg)<br>CV-AFS (for MeHg)	Hg:<br>2.66 ± 1.40<br>MeHg:<br>2.97 ± 1.45	MeHg:<br>3.67 ± 1.51	Significant differences in tHg levels in maternal blood between the second trimester, delivery, and 1 year postpartum.<br>Significantly higher tHg levels in maternal blood at one year postpartum than at delivery. Significantly higher tHg levels in maternal blood in the second trimester than at delivery.<br>Significantly higher MeHg levels in maternal blood one year after delivery than in the second trimester and at delivery.
Hansen et al., 2011 [40]	North Norwa y	211	2007-2009	Blood	ICP-MS	1.20	-	No increase in maternal blood Hg levels during pregnancy or from birth to 6 weeks postpartum. Consumption of fish, particularly shellfish, was a strong positive predictor of increased blood levels Hg.
Al-Saleh et al., 2014 [96]	Al-Kharj area, Saudi	1579	2005-2006	Blood	CV-AAS	1.95	2.87	Negligible relations of Hg levels in cord blood and in maternal blood to both placental thickness and

	Arabia							weight.
García-Esquinas et al., 2013 [61]	Spain	140	2003-2004	Blood	CV-AAS	3.90	6.72	Mercury levels were 25% higher in cord blood from mothers who consumed fish more often (>100 g/day). More than 70%

								of cord blood exceeded the Hg level of 5.80 μg/L.
Cabrera-Rodríguez et al., 2018 [55]	Canary Islands, Spain	471	2015-2016	Blood	ICP-MS		0.81 ± 0.56	No significant difference between cord blood Hg level with SGA (n = 47), AGA (n = 377), and LGA (n = 47).
de Assis Araujoet al., 2020 [39]	Rio de Janeiro, Brazil	140	2017-2018	Blood	ICP-MS	1.00	1.11	Significant positive correlation between Hg levels in maternal and cord blood.
Grandjean et al., 2005 [16]	Faroe Islands	1022	1986-1987	Blood	CV-AAS		22.35	Mercury levels in dry-weight cord were almost as good a predictor of MeHg-related neuropsychological deficits at age 7 as were cord blood Hg levels. Cord blood Hg analysis can be used as a valid measure of prenatal MeHg exposure.
Huang et al., 2017 [145]	Dong Gang, Taiwan	145	2010-2011	Blood	ICP-MS	3.60 ± 3.90	5.00 ± 7.30	Hg levels were about 1.4 times higher in cord blood than in maternal blood. Strong significant correlation (r_s = 0.76) between Hg levels in paired maternal/cord blood samples. Mothers who consumed higher amounts of seafood had about a 3-fold increased risk of high Hg blood

								levels<br>(>5.80 μg/L). Significant association of

								prenatal vitamin use (>3 times per week) with lower maternal Hg blood levels.
Huang et al., 2021 [23]	Sirajikhan and Pabna<br><br>Sadar Upazila, Bangladesh	745	2008-2011	Serum	ICP-MS		1.92 (1.35-2.79)	No significant association of cord blood Hg level with preterm birth.
Irwinda et al., 2019 [98]	Jakarta, Indonesia	51	2017	Serum	ICP-MS	2.33 (ND - 1.49)	3.50 (0.90-12.0)	No significant associations of Hg levels in maternal blood or cord blood with preterm birth.
Iwai-Shimada et al., 2019 [46]	Tohoku region, Japan	687	2001-2006	Blood	CVAAS (for tHg)<br>GC-ECD (for MeHg)	tHg:<br>5.42 (3.89-7.59)<br>MeHg: 5.15 (3.68-7.15)<br>iHg:	tHg:<br>9.96 (7.05-13.8)<br>MeHg: 9.47 (6.70-13.3)<br>iHg:	Total Hg and MeHg levels in cord blood were about twice as high as their levels in maternal blood. Strong correlations between both tHg levels and MeHg levels in maternal blood and in cord blood. Note: iHg levels were calculated by subtracting MeHg levels from tHg levels.

						0.24 (0.09-0.43)	0.27 (0.10-0.63)	
Rahbar et al., 2015 [44]	Kingston, Jamaica	100	2011	Blood	ICP-MS	-	4.00	No significant association of cord blood Hg levels with head circumference.
Rollin et al., 2009 [30]	Seven geographical areas, South Africa	96	2005-2007	Blood	ICP-MS	0.40 (0.18-0.82)	0.50 (0.10-5.40)	Significant correlation between Hg levels in maternal and cord blood. Mercury levels were higher in the two coastal populations (Atlantic and Indian Ocean sites). Significantly higher maternal Hg levels in the Atlantic population than in the other populations except the Indian Ocean population.
Sakamoto et al., 2010 [115]	Munakata City, Fukuoka, Japan	81	NP	Erythrocytes	CV-AAS	9.41 ± 4.19	15.3 ± 7.43	Significantly higher (~1.6-fold) Hg levels in cord erythrocytes than in maternal erythrocytes. Significant positive correlation between Hg levels in maternal and cord erythrocytes. Among Hg, Pb, As, Cd and Se, placental transfer of MeHg was extremely higher, while the transfer of Cd was limited. Protective effects of Se against MeHg were less pronounced in fetuses than in mothers.

Sekovanić et al., 2018 [164]	Zagreb and Zadar, Croatia	268	2008-2010	Blood	ICP-MS	2.40 ± 3.60	3.50 ± 6.40	No significant differences between Hg levels in either maternal blood or cord blood of smokers compared with controls.
Silver et al., 2018 [51]	Zhejiang Province, China	357	2008-2011	Blood	ICP-MS	-		Infants whose mothers worked outside the home had significantly higher levels of Hg in their cord blood and a greater chance of cord Hg exposure than infants whose mothers were housewives.
Soon et al., 2014 [165]	Honolulu, Hawaii	107	2010-2011	Blood	CV-AAS	-	5.20 ± 3.70	About 44% of participants had cord blood Hg levels of >5 μg/L. Significantly lower cord blood Hg levels in Filipina women than in non-Filipina women. Mercury levels did not differ by other demographic characteristics. Odds ratio for elevated Hg levels significantly higher among seafood consumers than among non-consumers
Tang et al., 2016 [81]	Shengsi Island, China	103	2011-2012	Serum	ICP-MS	-	23.4 ± 13.4	No significant associations between Hg exposure and birth weight, height, head circumference, or gestational age.

Dahiri et al., 2023 [42]	Seville, Spain	100	2020-2021	Blood	DMA-80 direct mercury analyzer	3.03	4.24	Significantly higher Hg levels in cord blood than in maternal blood. Strong correlation between Hg levels in these two blood compartments.

Gu et al., 2022 [31]	Peking, China	48	NP	Plasma	ICP-MS	0.73 (0.27-1.22)	0.82 (0.63-1.13)	No significant difference between Hg levels in maternal and cord blood.
Luo et al., 2024 [49]	Zhejiang province, China	48	2018-2019	Blood	ICP-MS	0.70 (0.60-1.10)	1.10 (0.80-2.10)	Positive correlation between Hg levels in maternal and cord blood.
Vigeh et al., 2006 [167]	Tehran, Iran	365	2003-2004	Blood	ICP-MS	1.34 ± 1.19	1.70 ± 1.33	No significant difference between cord blood Hg levels in PE cases and the control group.
Wang et al., 2022 [48]	Eight provinces of China	303 CHD cases and 303 healthy controls	2016-2020	Plasma	ICP-MS	3.03 (1.86-5.57)	-	Significantly higher blood Hg levels in cases with CHDs than in controls. Each unit of Hg level (μg/L) was related to a 2.88-fold increased risk of CHD after adjustment. Relation of Hg to an increased risk of both septal and conotruncal defects.
Yuksel et al., 2022 [120]	Turkey	41 IUGR cases and 34 controls	NP	Serum	CV-AAS	24.0 ± 7.13	24.7 ± 5.31	Significantly higher maternal serum and cord serum Hg levels in women with IUGR than in controls. Body weight, height, and head circumference were significantly lower when Hg levels were higher in maternal serum and cord serum. Mercury levels in cord serum could be

								used as a suitable biomarker for predicting low fetal

								weight, low fetal height, and low fetal head circumference.
Zhang et al., 2023 [168]	Shanghai, China	100	2013-2016	Blood	Direct Mercury Analyzer 80 (DMA-80)	1.39 ± 0.60	2.35 ± 1.28	Mercury levels were significantly lower during early, mid, and late pregnancy than before conception. Positive correlation between maternal blood Hg levels in late pregnancy and cord blood Hg levels of the newborn. Estimated placental transfer ratio for Hg was 1.68.

Abbreviations: *N* – total number of participants; NP – not presented; ND – not detected; LOD – limit of detection; iHg – inorganic mercury; MeHg – methyl mercury; tHg – total mercury; oHg – organic mercury; Zn – zinc, LC-PUFA – long-chain polyunsaturated fatty acids; Mn – manganese; Fe – iron; Ca – calcium; ICP-MS – inductively coupled plasma mass spectrometry; SF – sector field; HR – high resolution; AAS – atomic absorption spectrophotometry; CV – cold vapor; MAO – monoamine oxidase; GC-ECD – gas chromatography with an electron capture Detector; CV-AFS – cold vapor atomic fluorescence spectroscopy; PPROM – preterm premature rupture of membranes; NDTs – neural tube defects; SGA – small for gestational age; AGA – appropriate for gestational age; LGA – large for gestational age; PE – preeclampsia; GDM – gestational diabetes mellitus; IUGR – intrauterine growth restriction; CHDs – congenital heart defects.
